# Efficient removal of Sr²⁺, V⁵⁺, and Rb⁺ ions from groundwater using a hybrid Mg-MCM-41/Talc composite; Siwa Oasis in Egypt as case study

**DOI:** 10.1038/s41598-025-09553-3

**Published:** 2025-07-12

**Authors:** Hussein A. ELsayed, Mohamed Hamdy Eid, Umer Farooq, Ahmad Al-Qawasmeh, Abdehamid Albiad, Fahad Abdulaziz, Ahmed Mehaney, Péter Szűcs, Mostafa R. Abukhadra

**Affiliations:** 1 Department of Physics, College of Science, University of Ha’il, P. O. Box 2440, Ha’il, Saudi Arabia; 2 Institute of Environmental Management, Faculty of Earth Science, University of Miskolc, Miskolc- Egyetemváros, 3515 Hungary; 3 Materials Technologies and their Applications Lab, Geology Department, Faculty of Science, Beni-Suef University, Beni-Suef City, Egypt; 4 Department of Chemistry, College of Science, University of Ha’il, Ha’il, Saudi Arabia; 5 Physics Department, Faculty of Science, Beni-Suef University, Beni-Suef, 62512 Egypt; 6 Applied Science Research Center, Applied Science Private University, Amman, Jordan

**Keywords:** Mg-MCM-41/talc composite, Radioactive ions adsorption, Statistical physics modeling, Fixed-bed column, Groundwater remediation, Environmental chemistry, Physical chemistry

## Abstract

This study presents the development and application of a hybrid inorganic adsorbent composed of mesoporous Mg-MCM-41 integrated with exfoliated talc nanosheets (MCM/talc composite)—for the efficient removal of Sr²⁺, V⁵⁺, and Rb⁺ ions from contaminated water sources. The composite exhibited a mesoporous architecture (9.6 nm) and a specific surface area of 123.6 m²/g, combining the high reactivity of MCM-41 with the layered structure of talc. Batch adsorption experiments revealed exceptional saturation capacities (Q_sat_) of 229.9 mg/g (Sr²⁺), 188.8 mg/g (V⁵⁺), and 137.2 mg/g (Rb⁺). Kinetic data followed a pseudo-first-order model, while Langmuir isotherms confirmed monolayer adsorption. Critically, statistical physics modeling provided deep mechanistic insights into the adsorption process. The number of ions per active site (n) exceeded 2 for all ions, indicating multi-ionic vertical stacking at single adsorption sites. The adsorption energies (ΔE), derived from advanced monolayer modeling, were all below 8 kJ/mol. These values confirm a physisorption-dominant mechanism, governed by weak interactions such as van der Waals forces, hydrogen bonding, and electrostatic attractions. Fixed-bed column studies further validated the material’s dynamic performance, achieving removal efficiencies of 79.7% (Sr²⁺), 73.4% (V⁵⁺), and 68.6% (Rb⁺). Application to real groundwater from Egypt’s Siwa Oasis resulted in final concentrations of 1.3 mg/L (Sr²⁺), 0.46 mg/L (V⁵⁺), and 0.03 mg/L (Rb⁺) after two treatment cycles—meeting global health standards. This work demonstrates that the MCM/talc composite is a highly promising, low-cost, and reusable adsorbent for environmental remediation and selective recovery of critical metals, combining advanced theoretical modeling with field-relevant practicality.

**Supplementary Information:**

The online version contains supplementary material available at 10.1038/s41598-025-09553-3.

## Introduction

The contamination of water resources with hazardous chemicals poses a serious threat to environmental integrity and public health, destabilizing ecosystems and human communities^1^. A major contributor is the uncontrolled discharge of polluted effluents from mining, agriculture, and industrial activities^2,3^. These effluents release toxic elements into aquatic systems as free ions or complexed species, which persist, bioaccumulate, and may be carcinogenic^2,4–6^. Key contaminants include vanadium, boron, arsenic, zinc, barium, cobalt, rubidium, lead, and strontium^7–9^.

Strontium (Sr²⁺) is naturally present in groundwater and seawater at concentrations of 6–7 mg/L^10,11^. As an alkaline earth metal, it is widely used in glass, ceramics, pyrotechnics, and fluorescent lamps^10,12,13^. However, its radioactive isotope, strontium-90 (⁹⁰Sr), poses significant environmental and health risks. This highly soluble, beta-emitting radionuclide readily enters aquatic systems and accumulates through the food web, affecting human health^10,12,14,15^. Chronic Sr²⁺ exposure via drinking water has been linked to neurotoxicity, cognitive impairments, and developmental disorders^10,16,17^. Due to its similarity to calcium, Sr²⁺ can also replace it in bones, leading to skeletal deformities, bone cancer, and soft tissue malignancies^12,18^. Regulatory bodies such as the United States Environmental Protection Agency (EPA) and the World Health Organization (WHO) have set safe drinking water limits for Sr²⁺ between 1.5 and 4.2 mg/L^12^.

Vanadium is used in steel production, ceramics, batteries, glass, oil refining, pesticides, and medical technologies^19,20^. However, industrial discharge releases significant vanadium into aquatic systems, posing environmental and health risks. At elevated levels, vanadium is toxic and, when ingested via water or food, can cause respiratory issues, lipid peroxidation, paralysis, and damage to the liver, kidneys, nervous system, and digestive tract^21,22^. Of its oxidation states (− 1 to + 5), pentavalent vanadium (V⁵⁺) is the most stable, mobile, and toxic in the environment^23,24^. Linked to lung cancer and systemic toxicity, V⁵⁺ also disrupts microbial activity, plant growth, and water quality^25–27^. Although natural vanadium levels are usually low, most countries lack formal limits. Notably, the People’s Republic of China and the California Department of Public Health recommend a maximum of 0.05 mg/L in drinking water^23,28,29^.

Rubidium (Rb), a rare radioactive alkali metal, is used in electronics, energy storage, specialized glass, high-energy fuels, and medical technologies due to its high energy density, optoelectronic properties, and ease of ionization^30–32^. With declining natural reserves, alternative sources such as salt lake brines, groundwater, and seawater are gaining attention^33,34^. However, selective recovery of Rb⁺ is difficult due to the presence of competing ions^30,32^. Despite its industrial value, inorganic rubidium poses health risks if ingested through drinking water. Like uranyl, thorium, and cesium, it is toxic and potentially carcinogenic^35^. These radionuclides are particularly concerning due to their long half-lives, high solubility, and environmental persistence^36,37^. Their radiation can disrupt cell growth, cause genetic damage, and impair multiple systems, including reproductive, lymphatic, gastrointestinal, and vital organs such as the skin, eyes, heart, liver, kidneys, and central nervous system^36,37^.

The urgent need to control hazardous ion contamination in water systems has spurred interest in recovery methods that also enable industrial reuse, balancing health, economic, and environmental priorities. Several treatment methods—such as adsorption, flocculation, nanofiltration, biological degradation, membrane separation, ion exchange, and coagulation—have shown effectiveness^38–40^. Among these, adsorption stands out for its efficiency, low cost, and reusability in removing metal ions^41–43^. Advanced nanostructured adsorbents offer a practical, eco-friendly, and cost-effective solution for water purification^43,44^. Selection depends on factors like production cost, material availability, adsorption capacity, reusability, and selectivity^45,46^. Increasingly, research focuses on adsorbents from natural, low-cost materials—particularly rocks and minerals—due to their abundance, environmental compatibility, and scalability^47–49^.

Mesoporous silica nanoparticles are promising adsorbents for water treatment due to their high surface area and tunable pore structures^50,51^. Among them, MCM-type materials are notable for their uniform mesopores, thermal stability, and strong adsorption supported by silanol groups^52–55^. However, their commercial use is limited by high synthesis costs and toxic reagents^56^. To address this, natural silica-rich precursors have been explored as cost-effective, eco-friendly alternatives^56,57^. Research has mainly focused on aluminosilicate minerals like clay, while magnesium-rich phyllosilicates such as talc remain underused. Talc (Mg₃(Si₄O₁₀)(OH)₂), a naturally abundant mineral with a layered TOT structure and monoclinic symmetry, is low-cost, biocompatible, and has a high surface area^58–61^. Its integration with nanoporous phases like zeolites has been shown to enhance surface area, ion exchange, and adsorption capacity. Therefore, synthesizing mesoporous silica using exfoliated talc nanosheets presents a promising path toward advanced, efficient adsorbents^58,61^.

In this study, a hybrid magnesium-rich mesoporous silica material was synthesized by integrating MCM-41 structures with exfoliated talc nanosheets, and evaluated as an efficient adsorbent for the removal of Sr²⁺, V⁵⁺, and Rb⁺ ions from aqueous solutions. The adsorption behavior was analyzed using statistical physics-based equilibrium models, considering both steric parameters (e.g., saturation capacity, site occupancy) and energetic factors (e.g., adsorption energy, enthalpy, and entropy). The hybrid material’s dynamic performance was also assessed in a fixed-bed column system, focusing on breakthrough behavior for the targeted ions. Its practical applicability was demonstrated using groundwater samples from Siwa Oasis. Although stable Sr²⁺ and Rb⁺ ions were used, they are chemically representative of their radioactive counterparts (e.g., ⁹⁰Sr, ⁸⁶Rb), which exist in similar ionic forms in aqueous environments. Therefore, the findings are expected to extend to their radioactive analogs under comparable conditions.

## Site description and hydrology

**Fig. 1:**
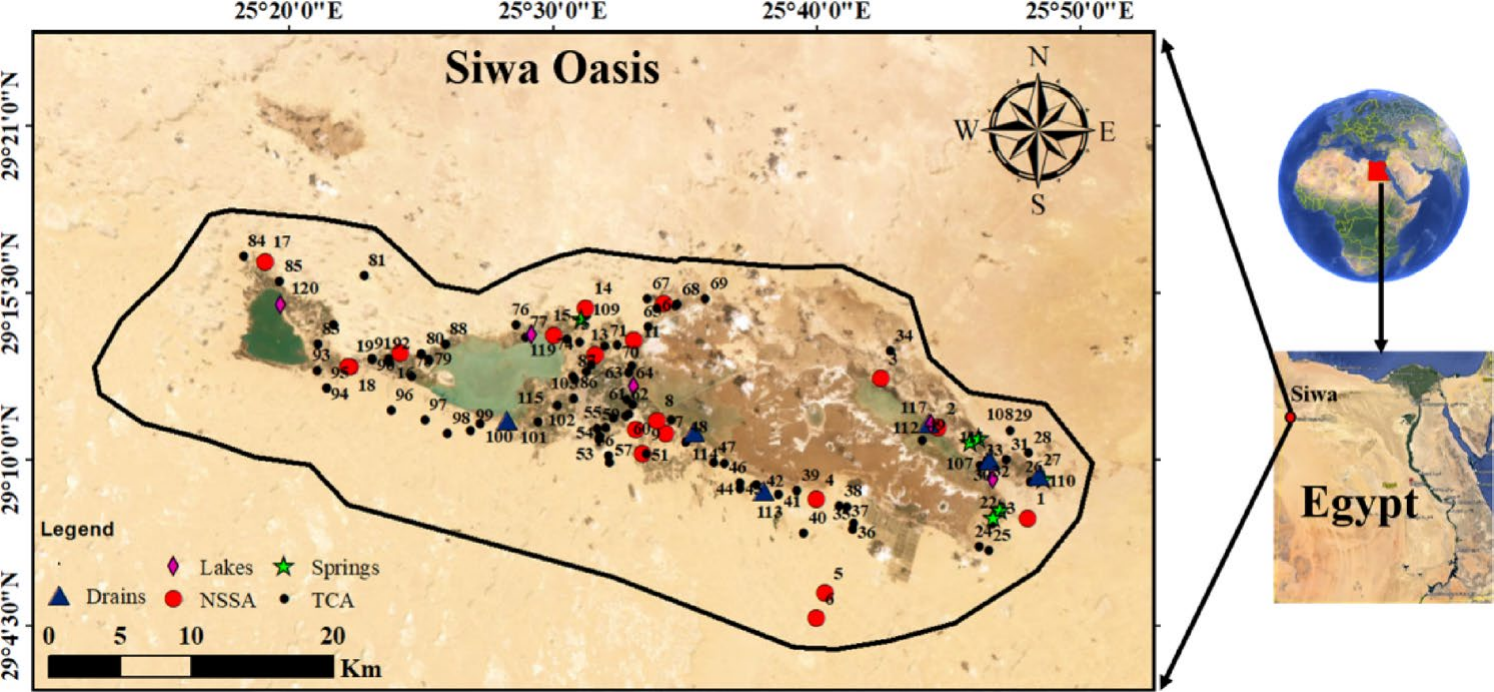
Location of sampling points including NSSA, TCA, springs, Drains and salt lakes (created using ArcGIS Pro 2.8.8 (Esri Inc., 2022; https://support.esri.com/en-us/patches-updates/2022/arcgis-pro-2-8-patch-8-2-8-8-announcement-8074*).*

Siwa Oasis, situated in Egypt’s northern Western Desert, spans an area of approximately 1,100 km² and lies around 320 km south of the Mediterranean coast (Fig. 1). The region supports an estimated population of 23,000 and sustains an economy largely dependent on agriculture, including palm cultivation, olive oil production, and the farming of fruits and vegetables. Supplementary industries such as mineral water bottling and olive oil extraction also contribute to local economic stability^62,63^. The oasis is characterized by a hyper-arid climate, receiving an average annual rainfall of only 10 mm, with high evaporation rates ranging from 5.4 mm/day in winter to 16.8 mm/day in summer. These harsh climatic conditions, compounded by the region’s geographic isolation, intensify pressure on limited water resources^64,65^.

Geologically, Siwa comprises a complex stratigraphy including surface Quaternary deposits, Miocene and Eocene formations of the Tertiary Carbonate Aquifer (TCA), and deeper Paleozoic and Mesozoic layers such as the Nubian Sandstone Aquifer (NSSA)^66^. The TCA is primarily utilized for agriculture and domestic use, while the NSSA serves as a key source for irrigation and drinking water^67,68^. However, excessive pumping has led to observable cones of depression and pressure declines, threatening aquifer sustainability. This hydrogeological context is integral to the current study, as groundwater samples collected from these aquifers were used to evaluate the real-world efficacy of the synthesized Mg-MCM-41/talc composite. The elevated levels of Sr²⁺, V⁵⁺, and Rb⁺ detected in these samples emphasize the urgent need for effective, site-adapted water treatment solutions.

## Experimental work

### Materials

The raw talc mineral used in this study was sourced from El-Nasr Mining Company, which extracts materials from the Bir Meseh area in the southern region of Shalatin, Eastern Desert, Egypt. A chemical analysis of the talc sample revealed its composition as follows: 46.35% SiO₂, 25.53% MgO, 9.54% CaO, 3.86% Al₂O₃, 2.16% Fe₂O₃, 0.13% P₂O₅, 0.02% K₂O, 0.28% TiO₂, 0.12% SO₃, 0.18% Na₂O, and 11.83% loss on ignition (L.O.I.).The materials employed for the synthesis process included cetyltrimethylammonium bromide (CTAB, 98%), ethanol (96%), and ammonium hydroxide (20% NH₃), all of which were procured from Sigma-Aldrich, Egypt. Additionally, standard solutions of strontium, vanadium, and rubidium, each with a concentration of 1000 mg/L, were supplied by Sigma-Aldrich and used to prepare the contaminated aqueous solutions for the study.

### Synthesis of Mg-MCM-14/exfoliated Talc composite (MCM/talc)

The synthesis of Mg-MCM-41 was carried out following established protocols outlined in^54^. Initially, raw talc samples were ground to achieve a particle size range of 25–100 μm. In a separate step, 0.5 g of the surfactant CTAB was dissolved by stirring in 50 mL of distilled water for 20 min. The resulting CTAB solution was combined with 16.34 mL of ammonia solution, and the mixture was stirred for an additional 20 min. Subsequently, 0.75 g of the ground talc powder was introduced into the solution under continuous stirring for 60 min. The mixture was then transferred to a Teflon-lined stainless steel autoclave, which was tightly sealed. The autoclave was heated at 110 °C for 24 h to facilitate the reaction. After the synthesis, the solid product was recovered through centrifugation at 3000 rpm. The obtained material was thoroughly washed with ethanol and distilled water to remove impurities and then dried at 80 °C for 10 h. The final product was stored for subsequent characterization and application studies.

### Characterization instruments

The crystalline structure and properties of the materials were characterized through X-ray diffraction (XRD) analysis using a PANalytical Empyrean diffractometer. The instrument enabled high-angle detection within the 2θ range of 0° to 70° and low-angle detection from 0° to 5°. The evolution of key chemical groups during the synthesis process was assessed with a Shimadzu FTIR-8400 S spectrometer, which operated across a wavelength range of 400 to 4000 cm⁻¹. The surface morphology of the synthesized materials was visualized using a Gemini Zeiss Ultra 55 scanning electron microscope. To enhance imaging quality, the samples were coated with a thin layer of gold prior to observation. Porosity and specific surface area measurements were obtained using a Beckman Coulter SA3100 surface area analyzer, following degassing of the samples. These analyses were conducted based on nitrogen (N₂) adsorption-desorption isotherms.

### Sampling and analysis of Raw water

A comprehensive water sampling campaign was carried out in February 2022 across the Siwa Oasis to ensure representative coverage of all regional water sources. A total of 110 strategically selected sampling locations were included, yielding 120 water samples from both deep and shallow aquifers—specifically, the Nubian Sandstone Aquifer (NSSA) and the Tertiary Carbonate Aquifer (TCA), which are the primary water sources for the area. Additional samples were collected from alternative sources, including six springs, six drainage channels, and five surface lakes. To maintain sample integrity, all collected water samples were stored in iceboxes at 4 °C until laboratory analysis. A detailed suite of physico-chemical analyses was conducted to assess water quality parameters. The sampling was conducted to evaluate the real contamination levels of Sr²⁺, V⁵⁺, and Rb⁺ in the Siwa Oasis groundwater and to demonstrate the practical applicability of the synthesized MCM-41/talc composite in treating real water samples.

The results revealed that numerous wells exhibited elevated levels of heavy metals, salinity, calcium and magnesium hardness, as well as chemical anions such as sulfate and nitrate—exceeding permissible limits for both drinking and irrigation use. Notably, strontium, vanadium, and rubidium were detected at concentrations significantly above international guideline values, with average levels recorded at 10.3 mg/L for strontium, 4.8 mg/L for vanadium, and 1.7 mg/L for rubidium. These elevated concentrations pose potential risks to environmental and public health, threatening aquatic ecosystems and human well-being. However, they also present a unique opportunity for resource recovery. Sr²⁺, V⁵⁺, and Rb⁺ are considered valuable strategic elements with wide-ranging industrial applications, highlighting the potential for their selective re-extraction and reuse from contaminated groundwater sources.

### Batch adsorption experiments

Batch adsorption experiments were carried out to examine the removal efficiency of Sr²⁺, V⁵⁺, and Rb⁺ ions using the synthesized MCM/talc material as an adsorbent. The adsorption experiments were conducted using individual (single-metal) aqueous solutions of Sr²⁺, V⁵⁺, and Rb⁺. These studies focused on assessing the effects of pH (2–7), initial ion concentrations (25–300 mg/L), and contact times (20–960 min). The experiments were conducted under standardized conditions, which included a solution volume of 100 mL, a temperature of 293 K, and an adsorbent dosage of 0.3 g/L. After equilibrium was reached, the solutions were filtered to separate the MCM/talc particles, and the remaining concentrations of Sr²⁺, V⁵⁺, and Rb⁺ were quantified. These measurements were performed using inductively coupled plasma mass spectrometry (ICP-MS, Perkin Elmer). Calibration standards for the metal ions were obtained from Merck (Germany) and validated by the National Institute of Standards and Technology (NIST). The data obtained were used to evaluate the adsorption performance of MCM/talc based on a mathematical Eq. 1. In this equation, the terms Q_e_​, C_o_​, C_e_​, V, and m represent adsorption capacity (mg/g), initial ion concentration (mg/L), equilibrium ion concentration (mg/L), solution volume (mL), and the mass of the adsorbent (mg), respectively:

1 $$\:{Q}_{e\:(mg/g)}=\frac{{(C}_{o}-{C}_{e})V}{m}$$

The adsorption behavior of Sr²⁺, V⁵⁺, and Rb⁺ onto MCM/talc was further analyzed using established kinetic models, equilibrium approaches, and advanced isotherm models informed by statistical physics (Table S1). Nonlinear regression techniques were applied to fit the experimental adsorption data. Performance metrics such as the coefficient of determination (*R*^*2*^) (Eq. 2) and the Chi-squared statistic (*χ*^*2*^) (Eq. 3) were used to evaluate model accuracy. Additionally, the root mean square error (RMSE) (Eq. 4) was calculated to assess the performance of equilibrium models in describing the adsorption data for Sr (II), V (V), and Rb (I). Here, Q_e, cal_​, Q_e, exp_​, and related terms correspond to calculated adsorption capacity, experimental adsorption capacity, and associated variables:

2 $$\:{\text{R}}^{2}=1-\frac{\sum\:({Q}_{e,\:exp}-{Q}_{e,\:cal}{)}^{2}}{\sum\:({Q}_{e,\:exp}-{Q}_{e,\:mean}{)}^{2}}\:$$

3 $$\:{{\upchi\:}}^{2}=\sum\:\frac{({Q}_{e,\:exp}-{Q}_{e,\:cal}{)}^{2}}{{\text{Q}}_{\text{e},\:\text{c}\text{a}\text{l}}}\:\:\:\:\:\:\:\:\:\:\:\:\:\:\:\:\:$$

4 $$\:\text{R}\text{M}\text{S}\text{E}=\sqrt{\frac{\sum\:_{\text{i}=1}^{\text{m}}({\text{Q}\text{i}}_{\text{c}\text{a}\text{l}}-{\text{Q}\text{i}}_{\text{e}\text{x}\text{p}}{)}^{2}}{{\text{m}}^{{\prime\:}}-\text{p}}}\:\:\:\:\:\:$$

### Fixed-bed column adsorption studies

A fixed-bed column setup was utilized to investigate the removal of Sr²⁺, V⁵⁺, and Rb⁺ ions from groundwater. This system comprised a borosilicate glass column (15 cm in length, 2 cm internal diameter) filled with MCM/talc powder as the adsorbent. The adsorbent was supported by polyethylene wool and a plastic mesh to prevent material loss. A peristaltic pump controlled the water flow rate through the column, which was maintained at a constant 5 mL/min. The initial pH (6) of the system was unchanged throughout the experiments, and the bed thickness varied between 1 cm and 3 cm. Effluent samples were collected at 60-minute intervals to monitor the removal efficiency.

Column performance metrics, such as breakthrough time, saturation time, removal efficiency, and breakthrough curve profiles, were evaluated. The definitions of breakthrough and saturation times corresponded to removal efficiencies of 10% and 95%, respectively. Additional operational parameters, including the treated effluent volume (V_eff​_), adsorbent capacity (C_ad​_), total adsorbed metal ions (Q_total​_), total introduced ions (M_total_​), column equilibrium capacity (Q_eq_​), and removal efficiency (R%), were calculated using the following equations:

5 $$\:{V}_{eff}=Q\:x\:{t}_{total}\:\:\:\:$$

6 $$\:{C}_{ad}={C}_{o}-{C}_{eff}\:\:\:\:\:$$

7 $$\:{q}_{total}\left(mg\right)=\frac{QA}{1000}=\frac{Q}{1000}{\int\:}_{t=0}^{t={t}_{total}}{C}_{ad}dt\:\:$$

8 $$\:{M}_{total}\left(mg\right)=\frac{{C}_{o}Q{t}_{total}}{1000}\:\:$$

9 $$\:{Q}_{eq}\left(mg/g\right)=\frac{{Q}_{total}}{X}\:\:$$

10 $$\:Total\:removal\:.,\%\:(R.,\%)=\frac{{Q}_{total}}{{M}_{total}}X\:100\:\:$$

Here, C_o_ and C_eff_ represent initial and effluent ion concentrations, Q is the flow rate, t_total​_is total operational time, A is the area under the breakthrough curve, and X is the mass of the MCM/talc adsorbent. This comprehensive analysis framework provided insights into the efficiency and adsorption characteristics of the column system.

## Results and discussion

### Characterization of the used adsorbent

**Fig. 2:**
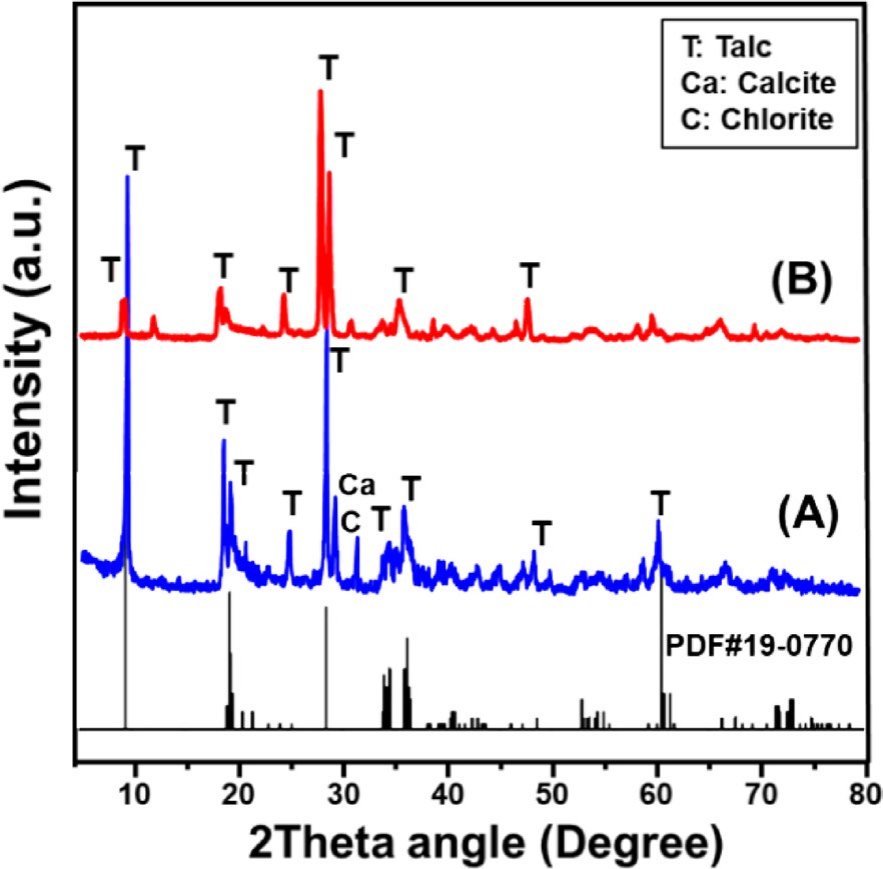
XRD patterns of raw talc **(A)** and synthetic MCM/expanded talc hybrid (MCM/talc) **(B)**.

The synthesis process was validated through X-ray diffraction (XRD) characterization, demonstrating the successful alteration of the pristine layered silicate structure of talc into an integrated hybrid composed of mesoporous silica and residual talc layers (Fig. 2). Initially, the XRD pattern of the untreated talc sample exhibited distinct reflections indicative of its mineralogical purity, predominantly as highly crystalline talc (PDF #29–1493), accompanied by minor mineral impurities including chlorite and calcite (PDF #00-005-0586), as shown in Fig. 2A^58,61^. Specifically, characteristic talc diffraction peaks appeared at 2θ positions around 9.48° (002), 19.21° (004), 28.62° (006), 36.31° (132), and 60.50° (−331), clearly delineating its monoclinic crystal structure and confirming the presence of typical interlayer spacing (d-spacing = 9.32 Å), consistent with the chemical composition Mg₃Si₄O₁₀(OH)₂ (PDF #19–0770; PDF #29-1493). Post-synthesis of the mesoporous material (MCM-41), substantial modifications were observed within the XRD diffraction profile, underscoring the significant influence exerted by synthesis conditions, chemical interactions, and the incorporation of the surfactant cetyltrimethylammonium bromide (CTAB). Most notably, as depicted in Fig. 2B, the intensity of the original talc reflections significantly decreased, highlighting the drastic structural disruption resulting from synthesis. Additionally, the residual peaks exhibited notable shifting toward lower diffraction angles, reflecting increased interlayer spacing and considerable structural rearrangement. These structural changes suggest a partial exfoliation or intercalation of CTAB molecules between the silicate layers, resulting in the loss of long-range crystalline order. Furthermore, the emergence of broadened and less intense peaks in the XRD pattern after synthesis directly indicates a transition toward mesoporosity, confirmed by a substantial reduction in crystallinity compared to the pristine talc.

**Fig. 3:**
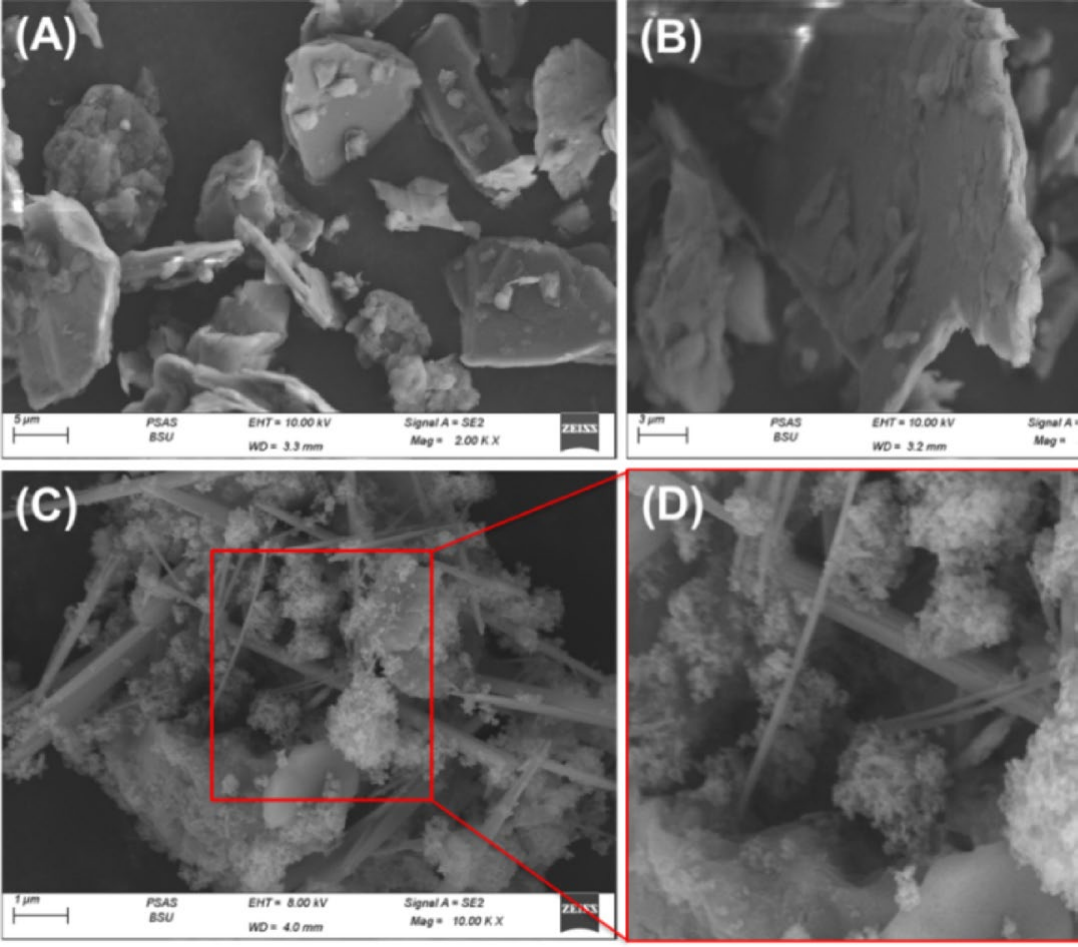
SEM images of raw talc (**A** and **B**) and synthetic MCM/talc particles (**C** and **D**).

The surface morphology of natural talc and the synthesized MCM-41/talc hybrid was investigated using scanning electron microscopy (SEM), as shown in Fig. 3. Images (A) and (B) represent the natural talc powder at magnifications of 2,000× and 3,000×, respectively. The micrographs reveal a characteristic lamellar, plate-like structure with relatively smooth and compact surfaces^58,61^. This morphology is typical of phyllosilicate minerals like talc, which consist of stacked silicate layers held together by weak van der Waals forces. However, after the formation of MCM-41 on the talc surface, as seen in images (3 C) and (3D), a substantial morphological transformation is evident. The talc surface becomes decorated with a dense array of fibrous and spherical meso-structures, indicating the successful deposition and in-situ growth of the mesoporous silica (MCM-41). These newly formed structures are well dispersed and intimately associated with the talc substrate, forming a hybrid network with significantly increased surface roughness and porosity. The porous nature and high surface area of MCM-41 are critical attributes that can enhance the physicochemical performance of the composite material. This hybridization is particularly significant for applications in catalysis, adsorption, and controlled release systems, where surface accessibility and functionalization are crucial. The intimate integration of MCM-41 onto talc not only preserves the structural stability of the base material but also imparts novel textural and surface properties that are desirable for multifunctional applications.

**Fig. 4:**
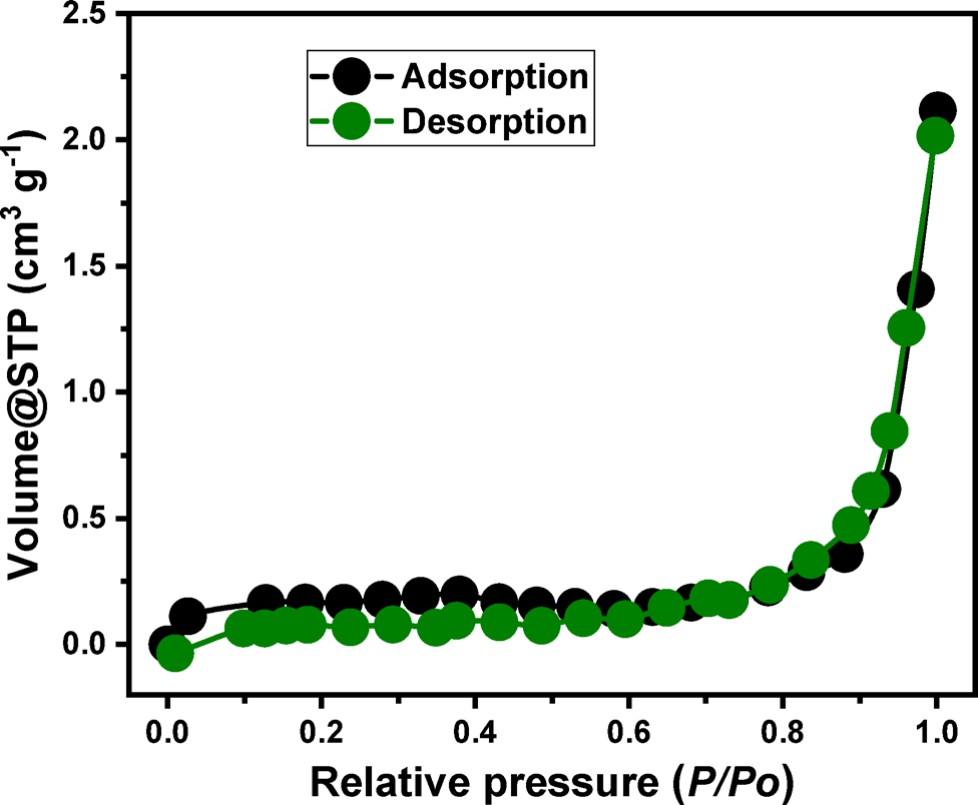
The nitrogen adsorption/desorption synthetic MCM/talc particles (C and D).

The nitrogen adsorption-desorption isotherm of the MCM/talc composite material is presented in Fig. 3. The observed isotherm corresponds closely to a Type IV isotherm based on the IUPAC classification, characteristic of mesoporous materials with pore diameters typically ranging between 2 and 50 nm (Fig. 4)^54^. A distinct hysteresis loop was observed, primarily within a relative pressure (P/P₀) range from approximately 0.7 to 1.0. The nature of the hysteresis loop is consistent with a Type H3 hysteresis, suggesting the presence of slit-like mesopores or voids resulting from the aggregation of plate-like particles or layered structures (Fig. 4). The sharp increase in adsorbed volume at high relative pressures (P/P₀ > 0.8) indicates capillary condensation phenomena within mesopores^58^. Furthermore, the absence of a defined plateau until approaching a relative pressure near unity highlights the broad pore-size distribution and potential presence of interparticle mesopores. Such mesoporosity is indicative of aggregated or stacked-layer structures, aligning with the anticipated morphology of the MCM/talc composite. This mesoporous structure, supported by the nitrogen adsorption-desorption characterization, underscores the potential applicability of the synthesized MCM/talc composite material in various adsorption applications, catalytic processes, and molecular separation technologies due to its enhanced surface area and pore accessibility. The determined avarge pore diamter was 9.6 nm confirming the mesoporous nature of the obtaiuned structure and the measured sufrace area was 123.6 m^2^/g highlighting its valubel qulaification as adsorbent.

### Batch adsorption studies

#### Effect of pH

**Fig. 5:**
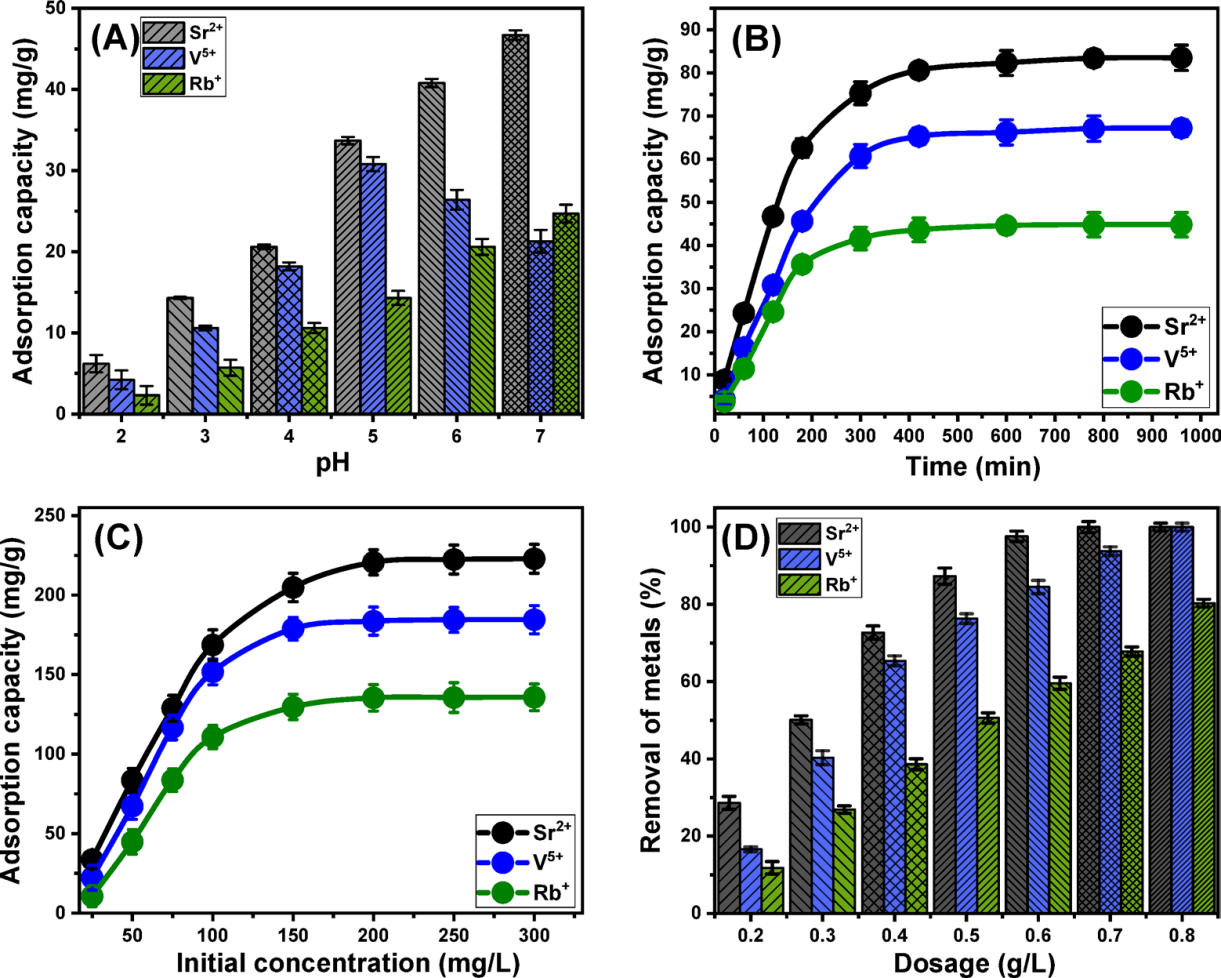
Show the influence of the experimental factors on the removal of the studied metals by MCM/talc including **(A)** pH, **(B)** contact time, **(C)** initial concentration, and **(D)** MCM/talc dosage.

The pH of an aqueous solution is a critical factor influencing the surface charge of adsorbents and the speciation of dissolved metal ions^69^. In this study, the impact of pH on adsorption performance was investigated within the range of 2 to 7, while maintaining constant experimental conditions: contact time of 120 min, temperature of approximately 20 °C, solution volume of 100 mL, initial metal ion concentration of 50 mg/L, and a MCM/talc dosage of 0.3 g/L. Adsorption capacities for Sr²⁺ and Rb⁺ exhibited a marked increase as pH rose, peaking at pH 7 with values of 46.7 mg/g for Sr²⁺ and 24.7 mg/g for Rb⁺ (Fig. 5A). For the uptake of V⁵⁺, the efficiency increased at remarkable rate up to pH5 (30.8 mg/g) and then declines clearly with the further increase in pH until pH 7 (Fig. 5A). This behavior can be attributed to changes in metal ion speciation and the deprotonation of surface functional groups on MCM/talc, which enhance its negative surface charge at higher pH levels. These results demonstrate that MCM/talc is an effective adsorbent across a pH range of 6 to 9, aligning with U.S. EPA guidelines for industrial wastewater treatment^70^, suggesting its potential for practical wastewater remediation applications.

Strontium predominantly exists as a divalent cation (Sr²⁺) in aqueous solutions across a pH range of 4 to 10, with negligible contributions from SrOH⁺ and SrCl⁺ species (Fig. S1)^70,71^. At elevated pH levels, the electrostatic attraction between Sr²⁺ ions and the negatively charged, deprotonated surface of MCM/talc becomes stronger due to the increased presence of hydroxyl groups on the adsorbent’s surface. In contrast, under acidic conditions, protonation and hydronium ion accumulation amplify electrostatic repulsion, reducing adsorption efficiency^72^. A similar mechanism applies to rubidium, which exists as a monovalent cation (Rb⁺) throughout the pH range studied (Fig. S1). Under alkaline conditions, the negatively charged MCM/talc surface facilitates electrostatic attraction with Rb⁺, while acidic conditions hinder this interaction due to surface protonation^32,73^. Vanadium in its pentavalent state (V⁵⁺) exhibits diverse ionic species depending on pH and concentration^24^. V⁵⁺ predominantly exists as the VO₂⁺ cation at pH < 3, while at pH > 4, it transitions to anionic forms such as H₂VO₄⁻, H₂V₂O₇²⁻, and HVO₄²⁻ (Fig. S1). The low adsorption observed at pH 2 and pH 9 may result from competitive interactions between VO₂⁺ and H⁺ ions at low pH, and between HVO₄²⁻ and OH⁻ ions at high pH, for available surface sites^24^.

To further elucidate the pH-dependent adsorption behavior, zeta potential measurements were conducted across a pH range of 2 to 9. The results revealed that the point of zero charge (pHₚzc) of the MCM/talc composite is approximately 4.5 for Sr^2+^ and Rb⁺ and 4.8 for V^5+^, indicating a shift in surface charge from positive (pH < pHₚzc) to negative (pH > pHₚzc). This surface behavior is critical in governing electrostatic interactions with metal ions. Specifically, the observed increase in adsorption efficiency for Sr²⁺ and Rb⁺ at higher pH can be attributed to enhanced electrostatic attraction to the negatively charged surface above the pHₚzc. In contrast, V⁵⁺ exhibits complex adsorption behavior due to pH-dependent speciation; it transitions from cationic VO₂⁺ at acidic pH to anionic vanadates (H₂VO₄⁻, HVO₄²⁻) above pH 4–5. The peak in V⁵⁺ adsorption at pH ≈ 5 coincides with a favorable charge balance between weakly negative surface sites and partially protonated vanadium species^74^. At higher pH values, electrostatic repulsion between the negatively charged surface and anionic vanadates explains the decline in V⁵⁺ uptake. These interpretations are fully supported by the combined analysis of speciation modeling (Figure S1) and zeta potential profiles (Figures S2), offering mechanistic validation for the distinct pH-responsive behavior of each ion. These findings emphasize the role of surface charge dynamics in controlling adsorption selectivity and provide a theoretical foundation for optimizing field-scale remediation conditions.

#### Effect of contact time

A detailed investigation was performed to evaluate the adsorption efficiency of MCM/talc for removing Sr²⁺, V⁵⁺, and Rb⁺ ions over a contact time ranging from 20 to 960 min. The study adhered to controlled conditions, including an initial ion concentration of 50 mg/L, a solution volume of 100 mL, a temperature of 20 °C, a pH of 7 (Sr²⁺and Rb⁺) and 5 (V⁵⁺), and an adsorbent dosage of 0.3 g/L. This setup aimed to assess the influence of contact time on the adsorption behavior of MCM/talc. The results demonstrated a marked enhancement in the adsorption of Sr²⁺, V⁵⁺, and Rb⁺ ions as the contact time increased, as evidenced by the amount of ions immobilized and the uptake rates (Fig. 5B). Adsorption efficiency showed rapid improvement during the initial phase and peaked at around 420 min. After this point, no further significant changes in adsorption rates or ion immobilization were observed, indicating that the system had reached equilibrium. The equilibrium adsorption capacities were determined to be 83.5 mg/g for Sr²⁺, 67.2 mg/g for V⁵⁺, and 44.8 mg/g for Rb⁺ (Fig. 5B).

The rapid adsorption observed in the early stages is likely due to the abundance of unoccupied and highly reactive adsorption sites on the MCM/talc nanoparticle surfaces. These sites facilitated efficient interaction and binding of Sr²⁺, V⁵⁺, and Rb⁺ ions, leading to significant initial uptake rates^9^. However, as the contact time increased, the progressive occupancy of these sites resulted in a gradual reduction in adsorption rates. This decrease is attributed to the exhaustion of active functional groups on the surface of MCM/talc, limiting further ion sequestration. During the later phases of adsorption, minimal changes in the adsorption capacity were observed, suggesting the system had reached a state of equilibrium. At this stage, the active sites of MCM/talc were fully occupied, preventing additional ion binding. This stabilization highlights the efficiency and predictability of MCM/talc as an adsorbent material for the specified ions under the given experimental parameters^36^. These findings emphasize the capability of MCM/talc to function as an effective adsorbent for the removal of Sr²⁺, V⁵⁺, and Rb⁺ ions. The rapid adsorption during the initial phase, followed by a stable equilibrium state, illustrates the material’s suitability for practical applications. Furthermore, the equilibrium data validate MCM/talc’s potential for ion removal processes, offering a reliable and efficient solution within the specified time frame.

#### Starting concentration

This study investigated how varying initial concentrations of Sr²⁺, V⁵⁺, and Rb⁺ ions influenced their maximum adsorption capacities and equilibrium conditions when MCM/talc was utilized as an adsorbent. The tested ion concentrations ranged from 25 to 300 mg/L, with other experimental conditions held constant, including a solution volume of 100 mL, a contact time of 24 h, an adsorbent dosage of 0.3 g/L, a pH of 6, and a temperature of 293 K. This design ensured that the effect of ion concentration on adsorption could be isolated and accurately assessed. The results indicated a strong positive correlation between the initial ion concentrations and the adsorption capacities of MCM/talc (Fig. 5C). As the initial concentrations increased, improvements were observed in ion diffusion rates and driving forces, which enhanced the transport and interaction dynamics between the ions and the active binding sites on the MCM/talc surface. These factors collectively led to a significant rise in the adsorption efficiencies of Sr²⁺, V⁵⁺, and Rb⁺^75^.

However, the trend of increasing adsorption efficiency with rising ion concentrations was observed only up to a specific threshold. Beyond this point, further increases in the initial concentrations of Sr²⁺, V⁵⁺, and Rb⁺ failed to improve the adsorption performance. This plateau effect is attributed to the saturation of active binding sites on the MCM/talc surface. At this stage, all functional sites capable of ion sequestration were fully occupied, preventing additional adsorption regardless of the concentration. Achieving the equilibrium state was critical for optimizing the adsorption process. The maximum adsorption capacities at equilibrium for Sr²⁺, V⁵⁺, and Rb⁺ were found to be 222.8 mg/g, 184.5 mg/g, and 135.7 mg/g, respectively (Fig. 4). These values underscore the exceptional performance of MCM/talc as an adsorbent. The observed adsorption behavior highlights the efficiency of MCM/talc in removing metal ions from solutions, particularly at elevated ion concentrations, until site saturation occurs. Furthermore, these findings emphasize the material’s suitability for practical applications. By demonstrating high adsorption capacities and robust performance under varying conditions, MCM/talc proves to be a promising solution for addressing the environmental contamination of these ions as well as their re-extraction.

#### Solid dosage

The impact of varying dosages of MCM/talc on the adsorption efficiency for Sr²⁺, V⁵⁺, and Rb⁺ ions was systematically examined within a dosage range of 0.2 g/L to 0.8 g/L. Experimental conditions were carefully controlled, including a solution volume of 100 mL, contact time of 24 h, initial ion concentration of 50 mg/L, pH of 6, and temperature maintained at 293 K. This approach ensured that the observed effects could be attributed solely to the changes in adsorbent dosage. The outcomes of this investigation are depicted in Fig. 5D, illustrating the relationship between adsorbent mass and removal efficiency for the selected ions. A progressive enhancement in removal efficiency was observed across all three ions as the MCM/talc dosage increased. This trend can be explained by the higher availability of active adsorption sites and the increased surface area provided by the larger quantities of adsorbent. These factors collectively facilitated more effective interactions between the ions and the functional groups on the adsorbent surface, thereby improving the adsorption performance^76^. However, beyond an adsorbent dosage of 0.6 g/L, the rate of improvement in removal efficiency began to plateau. This phenomenon indicates that the adsorption process reached its saturation point, where the active sites on MCM/talc were fully occupied. Consequently, further increases in the amount of adsorbent had minimal impact on the adsorption efficiency. Quantitatively, the removal efficiency for Sr²⁺ (50 mg/L) demonstrated a steady increase, starting at 28.6% with 0.2 g/L of MCM/talc, and reaching 50.1% (0.3 g/L), 72.7% (0.4 g/L), 87.3% (0.5 g/L), 97.6% (0.6 g/L), and 100% (0.7 g/L) (Fig. 5D). Similarly, the removal of V⁵⁺ rose from 16.6% at 0.2 g/L to 440.3% (0.3 g/L), 65.4% (0.4 g/L), 76.3% (0.5 g/L), 84.5% (0.6 g/L), 93.8% (0.7 g/L), and 100% (0.8 g/L). For Rb⁺, a comparable trend was observed, with efficiency increasing from 11.8% at 0.2 g/L to an impressive 80.3% at 0.8 g/L (Fig. 5D). This behavior highlights the importance of optimizing adsorbent dosage to balance cost-effectiveness with maximum adsorption performance, making MCM/talc a viable candidate for practical applications in environmental remediation and wastewater treatment as well as the extraction of such precious metals or elements.

#### Kinetic studies

##### Intra-Particle diffusion behavior

**Fig. 6:**
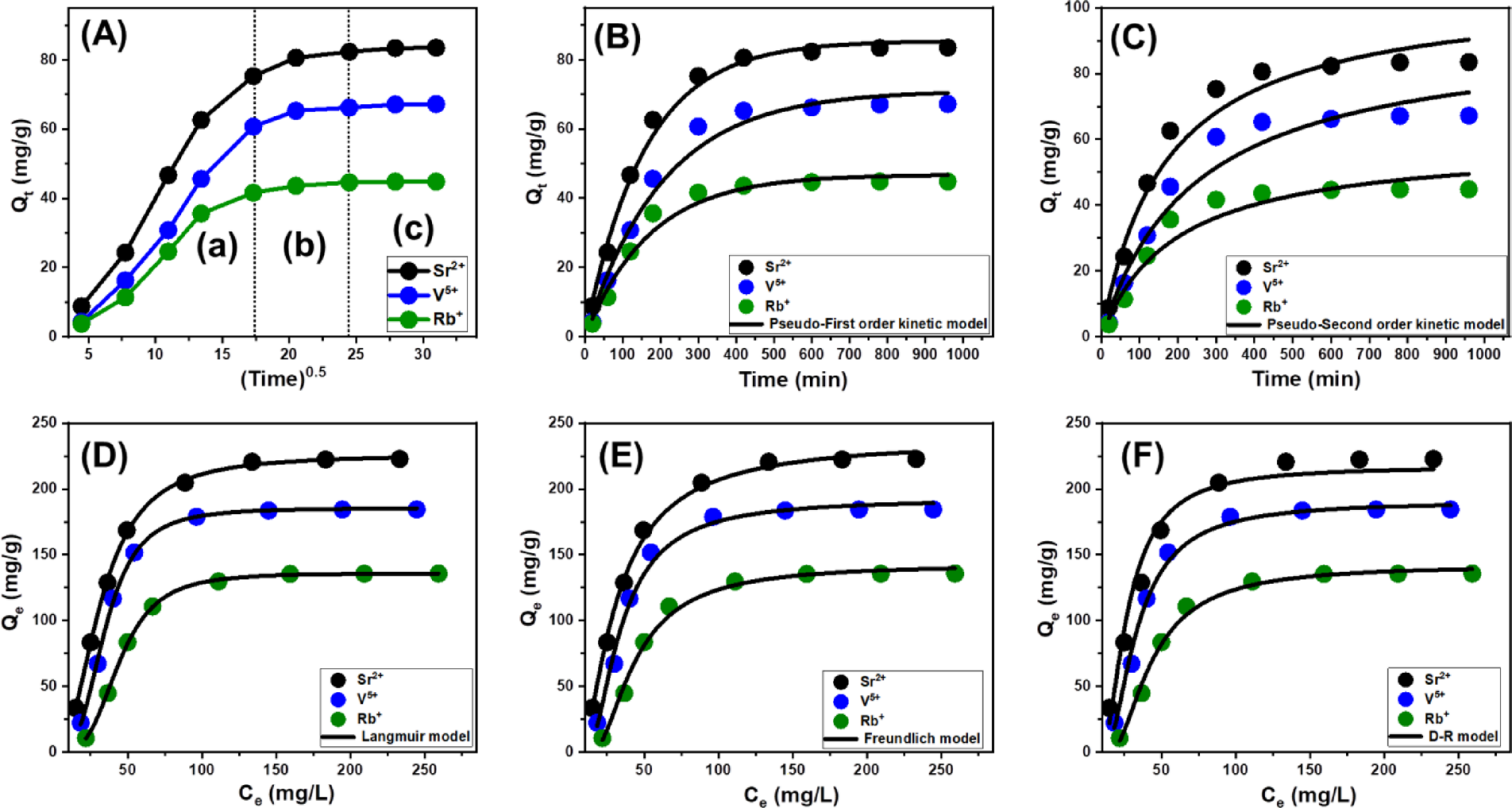
Show fitting of the adsorption behaviors of the studied metals with the different kinetic and isotherm models including **(A)** Intra-particle diffusion model (notice the different letters refer to the different segments in the curves), **(B)** Pseudo-First order kinetic model, **(C)** Pseudo-Second order kinetic model, **(D)** Langmuir isotherm model, **(E)** Freundlich isotherm model, and **(F)** D-R isotherm model.

The evaluation of intra-particle diffusion dynamics during the adsorption of Sr²⁺, V⁵⁺, and Rb⁺ ions onto MCM/talc provides a deeper understanding of the complex adsorption mechanisms and interaction pathways involved. The adsorption profiles depicted in Fig. 6A reveal three distinct regions with varying slopes, suggesting the presence of multiple adsorption stages and diffusion processes operating concurrently^77^. These stages can be divided into three primary phases:

A. Initial surface adsorption (External interactions): In the first phase, adsorption primarily occurs on the exterior surfaces of the MCM/talc particles. Metal ions readily interact with free, active receptor sites on the external framework of the adsorbent. This phase is crucial for efficient removal in the early stages of the adsorption process, as the abundance of surface-active sites facilitates rapid ion binding. The effectiveness of this surface-level interaction significantly contributes to the bulk of metal ion remediation during this stage (Fig. 6A).
B. Layered and internal adsorption (Diffusion and interior binding): As the surface adsorption sites become saturated, the process transitions into a secondary phase involving internal diffusion and layered adsorption. During this stage, metal ions penetrate the nanoporous structure of MCM/talc, binding to internal adsorption sites. The diffusion of ions from the surface into the inner layers of the adsorbent plays a dominant role in this phase. This progression not only extends the adsorption capacity but also establishes an additional pathway for ion removal within the internal structure of the adsorbent (Fig. 6A)^78,79^.
C. Equilibrium and saturation phase: In the final stage of the adsorption process, the system approaches a steady-state condition, where all available adsorption sites—including those on the surface and within the internal structure of the MCM/talc composite—have been completely utilized. At this point, the rate of ion uptake levels off, indicating that no further significant adsorption occurs via the initial mechanisms. The retention of metal ions is now maintained through weak molecular interactions and interionic forces, which prevent desorption but do not contribute to additional adsorption. This plateau phase signifies that the material has reached its maximum adsorption capacity, marking the completion of the adsorption cycle and confirming the full saturation of the MCM/talc adsorbent (Fig. 6A)^36^.

The study highlights the sequential nature of the adsorption process, beginning with external adsorption mechanisms that dominate during the initial phase. In this stage, the removal efficiency is largely dependent on the abundance of free surface-active sites. As these sites are progressively occupied, the adsorption process transitions to the internal adsorption phase, where diffusion and internal binding mechanisms drive metal ion removal. Finally, the equilibrium phase signifies full utilization of the MCM/talc’s binding sites, with adsorption stabilizing as molecular and interionic forces sustain ion retention. This systematic progression—from rapid surface adsorption to gradual internal diffusion and eventual equilibrium—illustrates the complex and multi-stage adsorption behavior of Sr²⁺, V⁵⁺, and Rb⁺ ions on MCM/talc.

##### Kinetic modeling

Understanding adsorption kinetics is fundamental to deciphering the physical and chemical processes that govern the adsorption of pollutants, particularly the interplay between mass transfer phenomena and chemical interactions that affect efficiency over time^80^. The kinetic behavior of Sr²⁺, V⁵⁺, and Rb⁺ ions during their adsorption onto MCM/talc was analyzed using the pseudo-first-order (PFO) and pseudo-second-order (PSO) models. These models offer valuable insights into the dynamics of adsorption and elucidate the mechanisms underlying the removal of these metal ions. The PFO model focuses on the relationship between the rate at which adsorption sites on MCM/talc are occupied and the total adsorption capacity, emphasizing systems driven by physical interactions. This model is particularly applicable to scenarios where van der Waals forces or electrostatic attractions dominate the adsorption process. Conversely, the PSO model highlights the role of chemical interactions, such as hydrogen bonding or covalent bonding, in influencing adsorption kinetics. By incorporating these complementary frameworks, the study provides a comprehensive analysis of the adsorption dynamics^80^.

To interpret the kinetic behavior of Sr²⁺, V⁵⁺, and Rb⁺ adsorption onto the MCM/talc composite, nonlinear regression analysis was conducted to compare how well the experimental data conformed to two commonly used kinetic models: the pseudo-first-order (PFO) and pseudo-second-order (PSO) equations. The coefficient of determination (R²) and chi-squared error (χ²) served as indicators of model accuracy and goodness-of-fit. Based on the outcomes presented in Table 1; Fig. 6A and C, the PFO model outperformed the PSO model, demonstrating a stronger alignment with the experimental data. This finding implies that physical adsorption mechanisms, primarily driven by electrostatic forces and van der Waals interactions, play a leading role in the ion uptake process. The theoretical equilibrium capacities predicted by the PFO model—85.4 mg/g for Sr²⁺, 71.1 mg/g for V⁵⁺, and 46.8 mg/g for Rb⁺—showed close agreement with the actual adsorption measurements, supporting the reliability of this model in capturing the dynamics of the system. Although the PSO model did not provide the best fit, it still offered a reasonable approximation of the observed behavior, indicating that chemical interactions, such as surface complexation or hydrogen bonding, may also contribute to the adsorption process but are likely secondary compared to physical forces^81,82^. This dual contribution underscores the complexity of the adsorption mechanism, where physisorption dominates while chemisorption provides supportive enhancement. These interactions may include hydrogen bonding or the formation of surface complexes, which enhance adsorption but do not dominate the overall mechanism. These findings are consistent with other studies that report the secondary role of chemical mechanisms in adsorption systems dominated by physical forces^81^.

The results also imply the potential formation of successive adsorption layers. In the initial phase, metal ions may chemically bind to the active sites on the MCM/talc surface, forming a primary adsorption layer. This initial chemical binding could subsequently promote the formation of additional adsorption layers through physical interactions, such as electrostatic attractions. This layered adsorption mechanism may contribute to the increased overall adsorption capacity of MCM/talc^83^. In summary, the adsorption kinetics of Sr²⁺, V⁵⁺, and Rb⁺ ions on MCM/talc are primarily governed by physical forces, as indicated by the superior fit of the PFO model to the experimental data. However, the secondary role of chemical interactions underscores the complexity of the adsorption process and highlights the potential for combined mechanisms to enhance adsorption capacity. These insights provide a robust framework for understanding and optimizing adsorption systems for the removal of metal ions.

#### Equilibrium studies

##### Classic isotherm models

Equilibrium studies are fundamental for understanding the behavior of adsorption systems, particularly the distribution of dissolved pollutants between the aqueous phase and the adsorbent under equilibrium conditions. These investigations provide valuable insights into the operational mechanisms and limitations of adsorption processes, helping optimize their performance. Equilibrium models are essential tools for elucidating three key aspects: (a) the adsorbent’s ability to selectively bind soluble ions at the adsorbent-solution interface, (b) the maximum quantity of ions that can be accommodated on the adsorbent’s surface, and (c) the overall adsorption capacity of the system^81,84^. In this study, the equilibrium behavior of Sr²⁺, V⁵⁺, and Rb⁺ ions on MCM/talc was evaluated using three widely applied adsorption isotherm models: the Langmuir model (Fig. 6D), the Freundlich model (Fig. 6E), and the Dubinin–Radushkevich (D–R) model (Fig. 6F). Nonlinear regression analysis was employed to fit the experimental data to these models and to compare the predicted equilibrium parameters with experimental observations. The models’ applicability and accuracy were determined based on correlation coefficients (R²) and chi-squared (χ²) values (Table 1).

The Langmuir model demonstrated the best fit to the experimental data, as indicated by its superior R² and χ² values (Table 1). This suggests that the adsorption of Sr²⁺, V⁵⁺, and Rb⁺ ions onto MCM/talc occurs on a homogeneous surface where all binding sites exhibit uniform affinity for the adsorbate^82,84^. Consistent with the Langmuir model’s assumption, the adsorption process involves the formation of a monolayer of adsorbate ions evenly distributed over the adsorbent’s surface^77^. Furthermore, the dimensionless separation factor (R_L_) values were less than 1, indicating favorable adsorption conditions^85^. The theoretical maximum adsorption capacities (Q_max_) derived from the Langmuir model were 226.1 mg/g for Sr²⁺, 185.6 mg/g for V⁵⁺, and 136.1 mg/g for Rb⁺ (Table 1). These results emphasize the high efficiency of MCM/talc in adsorbing these metal ions.

**Table 1:** The theoretical parameters of the studied the kinetic models, classic isotherm, and advanced isotherm models.

Model	Parameters	Sr²⁺	V⁵⁺	Rb⁺
Kinetic models				
Pseudo-first-order	***K***_***1***_ **(min**^**−1**^**(**	0.0063	0.0048	0.0058
	***Qe***_***(Cal)***_ **(mg/g)**	85.4	71.1	46.8
	***R*** ^***2***^	0.99	0.98	0.98
	***X*** ^***2***^	0.15	0.41	0.31
Pseudo-second-order	**k**_**2**_ **(g mg**^**−1**^ **min**^**−1**^**)**	5.65 × 10^−5^	4.41 × 10^−5^	8.79 × 10^−5^
	***Qe***_***(Cal)***_ **(mg/g)**	106.1	93.1	59.3
	***R*** ^***2***^	0.97	0.97	0.96
	***X*** ^***2***^	0.57	0.84	0.64
Classic isotherm models				
Langmuir	***Q***_***max***_ **(mg/g)**	226.1	185.6	136.1
	***b*** **(L/mg)**	3.4 × 10^−4^	1.08 × 10^−5^	1.49 × 10^−6^
	***RL***	0.99	0.99	0.99
	***R*** ^***2***^	0.99	0.99	0.99
	***X*** ^***2***^	0.024	0.066	0.015
Freundlich	***1/n***	0.59	0.46	0.45
	***k***_***F***_ **(mg/g)**	236.1	192.3	142.7
	***R*** ^***2***^	0.98	0.98	0.98
	***X*** ^***2***^	1.68	0.48	0.35
D-R model	***β*** **(mol**^**2**^**/kJ**^**2**^**)**	0.0054	0.0071	0.0076
	***Q***_***m***_ **(mg/g)**	226.9	190.3	142.3
	***R*** ^***2***^	0.97	0.99	0.99
	***X*** ^***2***^	1.45	0.45	0.32
	***E*** **(kJ/mol)**	9.26	8.39	8.11
Advanced isotherm model				
Monolayer model of one energy site	***R*** ^***2***^	0.99	0.99	0.99
	***X*** ^***2***^	0.024	0.066	0.015
	***n***	2.35	3.22	3.54
	***Nm*** **(mg/g)**	97.86	58.65	38.75
	***Q***_***sat***_ **(mg/g)**	229.9	188.8	137.2
	***C1/2*** **(mg/L)**	31.69	34.67	44.23
	***ΔE*** **(kJ/mol)**	−7.47	−4.53	−6.99

The Dubinin–Radushkevich (D–R) isotherm model offers valuable understanding of the energy dynamics governing the adsorption process. In contrast to conventional isotherm models, the D–R model emphasizes the calculation of mean free energy (E), which is a key parameter for distinguishing the nature of the adsorption interactions—whether they are primarily physical, chemical, or a combination of both^86^. Based on the magnitude of E, one can deduce the type of interaction taking place: values below 8 kJ/mol typically signify physisorption, dominated by weak, non-covalent forces; values between 8 and 16 kJ/mol reflect a mixed mechanism, where both physical and chemical processes are involved; and values exceeding 16 kJ/mol are indicative of chemisorption, usually involving stronger, more specific bonds like covalent attachments^86,87^. In the case of Sr²⁺, V⁵⁺, and Rb⁺ adsorption onto the MCM/talc composite, the calculated E values fall within the intermediate range of 8–16 kJ/mol (as shown in Table 1), suggesting that the process is governed by a hybrid mechanism. At the onset, adsorption is primarily facilitated by non-specific interactions such as van der Waals forces and electrostatic attractions, which allow for quick and reversible ion attachment. As adsorption progresses, chemical interactions—potentially involving surface complexation or hydrogen bonding—begin to contribute, thereby enhancing both the strength and retention of adsorbed ions. This synergistic adsorption mechanism highlights the functional versatility of the MCM/talc composite, offering both high affinity for target pollutants and regenerability, making it highly suitable for repeated cycles of application in environmental remediation.

The findings from the equilibrium studies highlight the versatility and efficiency of MCM/talc as an adsorbent for the removal of Sr²⁺, V⁵⁺, and Rb⁺ ions. The better performance of the Langmuir model suggests that monolayer adsorption is the dominant mechanism, with all binding sites being equally accessible to the ions. The contribution of the D–R model reveals the energetic interplay between physical and chemical interactions, emphasizing the adaptability of MCM/talc in pollutant remediation.

##### Advanced isotherm models

**Fig. 7:**
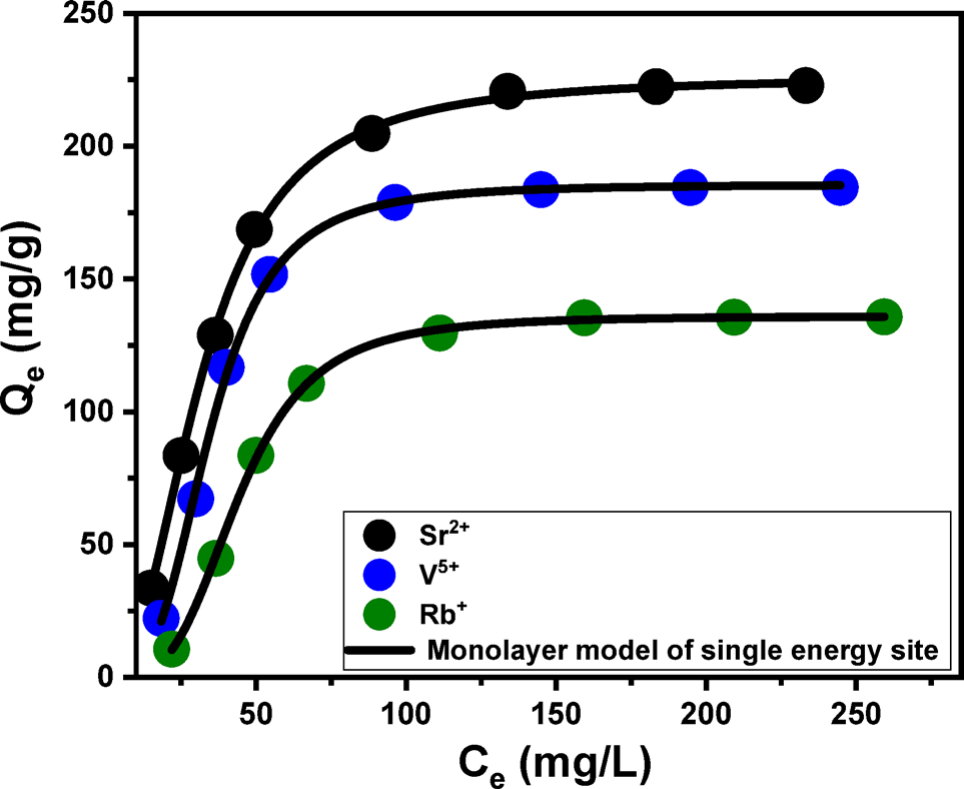
Non-linear fitting results of the uptake results with advanced Monolayer model of single energy site.

To gain a deeper understanding of the adsorption equilibrium behavior, a statistical physics-based modeling framework was employed. This theoretical approach provides a microscopic insight into how pollutants interact with specific functional groups on the surface of the adsorbent, which act as binding or receptor sites. The model integrates steric and energetic parameters through advanced computational simulations, allowing for a detailed evaluation of the adsorption dynamics. Among the critical variables analyzed were the density of active adsorption sites (Nm), the average number of ions attached per site (n), the adsorption energy (ΔE), and the saturation adsorption capacity (Q_sat_) for Sr²⁺, V⁵⁺, and Rb⁺ ions. To ensure accurate fitting of the model to experimental data, the Levenberg–Marquardt optimization algorithm was applied in conjunction with multivariable nonlinear regression techniques (as shown in Fig. 7; Table 1). This modeling approach is grounded in the assumption that each receptor site is energetically uniform and acts independently, meaning that adsorbed ions do not significantly influence neighboring sites. Overall, this methodology allowed for a nuanced interpretation of both the spatial distribution of ions and the energetic landscape of the adsorption process on the MCM/talc surface.

The parameter n was pivotal in understanding the spatial arrangement of metal ions on the MCM/talc surface. When *n* < 1, ions are adsorbed in a horizontal configuration, indicating limited stacking and a multi-docking mechanism dominated by weak intermolecular forces. Conversely, *n* > 1 suggests vertical or multi-layered arrangements, where individual adsorption sites accommodate multiple ions. This vertical alignment reflects stronger ion-surface interactions and multi-ionic mechanisms that enhance adsorption efficiency^88,89^. The values of n for Sr²⁺, V⁵⁺, and Rb⁺ were 2.35, 3.22, and 3.54, respectively, indicating the capacity of single adsorption sites to hold multiple ions (Table 1). These results highlight the role of multi-ionic interactions in driving the adsorption of the three metals, predominantly in vertical orientations.

The adsorption capacity per site varied among the metals, influenced by factors such as ionic radius, charge density, and mobility. Each receptor site on MCM/talc could adsorb up to 3 Sr²⁺ ions and 4 ions of V⁵⁺ and Rb⁺, reflecting differences in the aggregation tendencies of these metals. Rb⁺ demonstrated the highest aggregation potential, attributed to a pronounced multi-docking effect. The total number of occupied adsorption sites (*N*_*m*_​) was determined to be 97.8 mg/g for Sr²⁺, 58.6 mg/g for V⁵⁺, and 38.7 mg/g for Rb⁺, consistent with the aggregation properties and experimentally observed adsorption capacities. The saturation uptake capacity (*Q*_*sat*​_) depended on both the site density (Nm​) and the number of ions adsorbed per site (n), with estimated values of 229.9 mg/g for Sr²⁺,188.8 mg/g for V⁵⁺, and 137.2 mg/g for Rb⁺ (Table 1). These results underscore the role of MCM/talc’s structural features in optimizing adsorption performance.

The adsorption energy (ΔE) provided crucial insights into the nature of the adsorption mechanisms. Physisorption processes typically exhibit binding energies below 40 kJ/mol, while chemisorption is associated with higher energies, often exceeding 80 kJ/mol. The *ΔE* values were calculated using Eq. 5, which incorporates the solubility (*S*), the gas constant (R), concentrations at half-saturation (C1/2), and system temperature (*T*)^90^:

5 $$\:\varDelta\:E=RT\:ln\left(\frac{S}{C}\right)\:\:\:\:\:\:$$

**Fig. 8:**
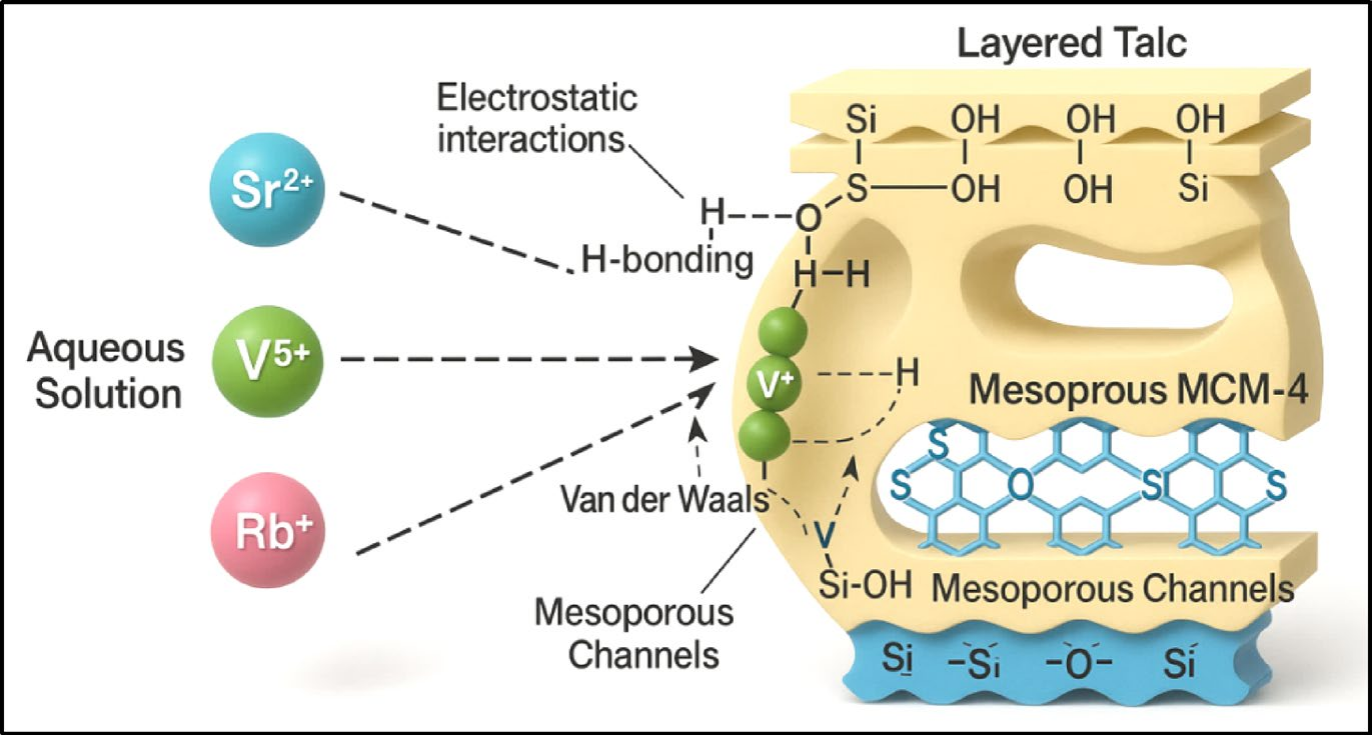
Schematic diagram for the adsorption mechanism of the studied metal into into the structure of MCM/talc.

The *ΔE* values for Sr²⁺, V⁵⁺, and Rb⁺ were all below 8 kJ/mol, confirming that physical interactions dominate the adsorption process (Table 1). Specific forces involved include: (A) hydrogen bonding (ΔE < 30 kJ/mol) which likely arising from interactions between hydroxyl groups on the MCM/talc surface and electronegative atoms in the metal ions^90,91^, (B) electrostatic attractions (ΔE = 2–50 kJ/mol): resulting from charge-based interactions between metal ions and the adsorbent surface^90^, (C) Van der Waals forces (ΔE = 4–10 kJ/mol), and (D) dipole-dipole interactions (ΔE = 2–29 kJ/mol): contributing to the stability of adsorption (Fig. 8). The negative ΔE values confirmed the exothermic nature of the adsorption process, highlighting its spontaneity and thermodynamic favorability. These findings suggest that the weak binding energies associated with physisorption are advantageous for practical applications, as they facilitate efficient desorption and enable adsorbent regeneration. The relatively weak binding energies allow for the reversible adsorption of metal ions, enhancing the material’s reusability and economic viability. Additionally, the multi-ionic interaction mechanisms contribute to the high adsorption capacity and efficiency, making MCM/talc a promising candidate for large-scale environmental remediation efforts. The adaptability of MCM/talc in accommodating various metal ions underscores its potential for diverse applications in pollutant management.

#### Recyclability and regeneration

The potential of the MCM/talc composite for practical and commercial-scale water treatment applications is closely linked to its ability to retain performance over multiple adsorption–desorption cycles. To assess this, a regeneration protocol was applied to previously used adsorbent samples, involving an initial rinsing step using distilled water under agitation with an orbital shaker at room temperature for 30 min. Following this, the samples were further washed, sterilized using distilled water, and dried at 50 °C for 12 h to restore their structural and functional integrity. The recyclability performance was evaluated through successive adsorption cycles using a standard set of operating parameters: an initial metal concentration of 50 mg/L, adsorbent dosage of 0.8 g/L, total volume of 100 mL, pH 6, contact time of 24 h, and a temperature of 293 K. The removal efficiencies for Sr²⁺, V⁵⁺, and Rb⁺ were systematically monitored across five consecutive cycles to assess the durability and sustainability of the composite under repeated use.

The composite demonstrated remarkable retention of adsorption capacity, particularly for Sr²⁺ and V⁵⁺. The removal efficiency for Sr²⁺ was maintained at 100% during the first two cycles, followed by a slight decline to 97.6%, 95.2%, and 90.4% in cycles 3 to 5, respectively. For V⁵⁺, a similar trend was observed, with initial removal at 100% dropping progressively to 95.4%, 91.3%, 88.7%, and 82.6% over the same cycles. In the case of Rb⁺, a moderate but steady decrease was noted, with removal efficiencies of 80.3%, 78.5%, 72.6%, 70.3%, and 65.7% from the first through the fifth cycle. The gradual reduction in adsorption efficiency across cycles is attributed to the cumulative saturation of active binding sites and potential formation of stable metal–ligand complexes at the adsorbent surface. These phenomena may reduce the number of available functional groups and limit the regeneration capacity over time, particularly in the case of ions that form stronger or less reversible interactions with the surface. These results underscore the promising reusability of the MCM/talc composite, especially for Sr²⁺ and V⁵⁺, where high removal efficiencies are retained over multiple cycles. While some decline in performance is expected due to surface saturation and possible structural fatigue, the composite still maintains a functional level of activity after five cycles, which is encouraging for real-world application. In future work, post-adsorption XRD and SEM analyses will be conducted to evaluate potential structural and morphological changes in the MCM/talc composite after repeated adsorption–desorption cycles. This will provide further confirmation of the material’s long-term stability, surface integrity, and suitability for practical environmental applications.

### Fixed bed column studies

#### The experimental performance of the mcm/talc bed

**Fig. 9:**
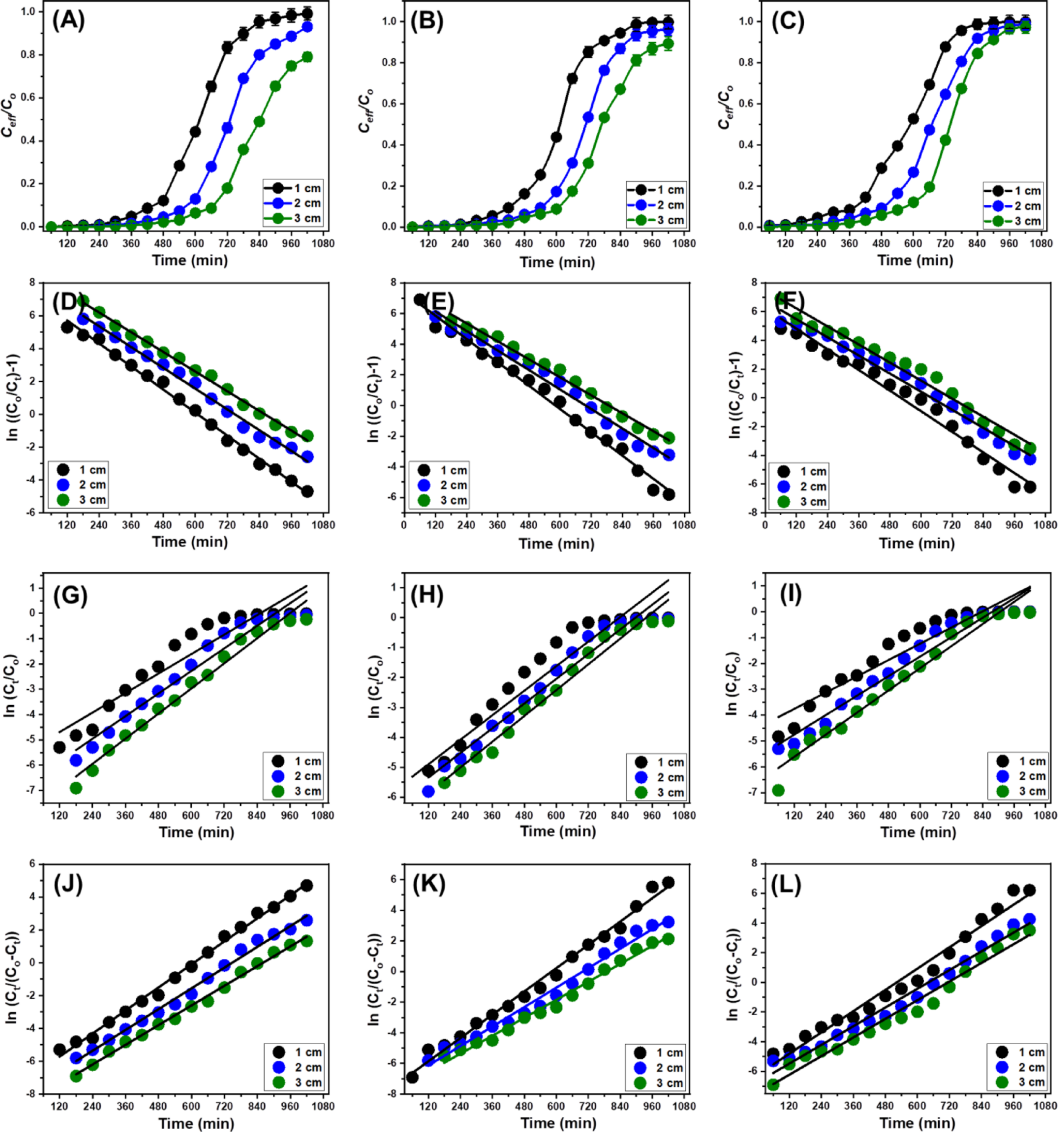
Breakthrough and model fitting curves for the adsorption of Sr²⁺, V⁵⁺, and Rb⁺ ions onto the MCM/talc composite under fixed-bed column conditions. Panels (**A**–**C**) show the breakthrough curves for Sr²⁺ (A), V⁵⁺ (B), and Rb⁺ (C), while panels (**D**–**F**) present the corresponding Thomas model fittings. Panels (**G**–**I**) illustrate the Adams–Bohart model fittings for Sr²⁺, V⁵⁺, and Rb⁺, respectively, and panels (**J**–**L**) display the Yoon–Nelson model fittings for each ion.

The influence of MCM/talc bed thickness on the adsorption efficiency of column systems designed for Sr²⁺, V⁵⁺, and Rb⁺ removal was systematically assessed across thicknesses ranging from 1 to 3 cm (Fig. 9). The experiments were carried out under controlled conditions, including a pH of 6, an initial metal ion concentration of 10 mg/L, a flow rate of 5 mL/min, and a total operation time of 1020 min. The findings demonstrated that increasing the bed thickness significantly enhanced both the operational lifespan and the decontamination efficacy of the column for all tested metals (Fig. 9A, B, and C; Table 2). For Sr²⁺ adsorption, breakthrough times increased markedly with thicker MCM/talc beds, reaching 480 min, 600 min, and 720 min for 1 cm, 2 cm, and 3 cm beds, respectively (Fig. 9A; Table 2). Exhaustion times exceeded 1020 min for the 3 cm bed. The removal efficiency of Sr²⁺ also improved with increasing bed thickness, with removal rates of 56.9% for 1 cm, 69.4% for 2 cm, and 79.7% for 3 cm after treating approximately 5 L of polluted water. Optimal performance was observed with a 3 cm bed, where the total Sr²⁺ ions introduced into the column (M_total​_) were 459 mg, of which 305.5 mg were adsorbed (Q_total_​). This corresponded to a bed adsorption capacity (C_ad_​) of 135.4 mg and a maximum adsorption capacity (Q_eq_​) of 81.9 mg/g for MCM/talc under the given conditions (Fig. 9A; Table 2).

Comparable trends were observed for V⁵⁺ (Fig. 9B; Table 2) and Rb⁺ (Fig. 9C; Table 2) adsorption. With a 3 cm bed, breakthrough times were recorded at 660 min for V⁵⁺ and 600 min for Rb⁺, while exhaustion times extended beyond 1020 min for V⁵⁺ and 960 min for Rb⁺. The removal efficiencies were 73.4% for V⁵⁺ and 68.6% for Rb⁺ after processing approximately 5 L of contaminated water. The total metal ions introduced into the column (M_total​_​) were 459 mg for both metals, with total adsorbed amounts (Q_total_​) of 262.9 mg for V⁵⁺ and 230.1 mg for Rb⁺. This yielded bed adsorption capacities ((C_ad_​) of 124.9 mg/g for V⁵⁺ and 116.7 mg/g for Rb⁺, with corresponding maximum adsorption capacities (Q_eq_​) of 43.8 mg/g for V⁵⁺ and 38.3 mg/g for Rb⁺ (Table 2). The improved adsorption performance observed with increased bed thickness can be attributed to a reduction in axial dispersion during mass transfer, which facilitates more effective diffusion of metal ions into the MCM/talc particles^92^. Additionally, thicker beds extend the residence time of metal ions within the column, allowing prolonged interaction with active adsorption sites and thereby enhancing metal ion capture efficiency^93^. This synergistic effect of minimized axial dispersion and increased residence time highlights the importance of optimizing bed thickness to improve the adsorption performance of column systems for heavy metal removal.

**Table 2:** The mathematical parameters of the obtained breakthrough curves and the investigated dynamic models.

Mathematical parameters																														
Bed height	**Flow rate**				**Conc.**,				**C**_**ad**_ **(mg/L)**		**Q (**_**total**_ **)** **(mg)**			**Q**_**eq**_ **(mg/g)**						**M**_**(total)**_ **(mg)**			**R.**,**%**	**t**_**b**_ **(min)**			**t**_**s**_ **(min)**		**V**_**eff**_ **(mL)**	
Aqueous solutions																														
Sr²⁺																														
1 cm	**5 mL/min**				**5 mg/L**				96.8		163.9			81.9						459			56.9	480			840		4200	
2 cm	**5 mL/min**				**5 mg/L**				117.9		236.4			59.1						459			69.4	600			1020		5100	
3 cm	**5 mL/min**				**5 mg/L**				135.4		305.5			50.9						459			79.7	720			-------		5100	
V⁵⁺																														
1 cm	**5 mL/min**				**5 mg/L**				95.3		158.8			79.4						459			56.1	480			840		4200	
2 cm	**5 mL/min**				**5 mg/L**				112.5		215.6			53.9						459			66.2	540			960		4800	
3 cm	**5 mL/min**				**5 mg/L**				124.9		262.9			43.8						459			73.4	660			-------		5100	
Rb⁺																														
1 cm	**5 mL/min**				**5 mg/L**				88.9		142.5			71.2						459			52.3	360			780		3900	
2 cm	**5 mL/min**				**5 mg/L**				105.4		192.2			48.1						459			61.9	480			900		4500	
3 cm	**5 mL/min**				**5 mg/L**				116.7		230.1			38.3						459			68.6	600			960		4800	
Real water																														
3 cm	**5 mL/min**				**Sr²⁺**				74.8		101.7			16.9						472.7			42.7	300			720		3600	
3 cm	**5 mL/min**				**V⁵⁺**				45.9		78.4			13.1						220.3			56.3	420			840		4200	
3 cm	**5 mL/min**				**Rb⁺**				19.5		39.3			6.55						78.03			67.4	480			-------		5100	

Kinetic model																														
Parameters							**Thomas Model**										**Adams–Bohart**								**Yoon-Nelson model**					
Thickness				**Flow rate**			**R** ^**2**^	**K**_**Th**_ **((L/h·mg)**				**Q**_**o**_ **(mg/g)**					**R** ^**2**^	**K**_**AB**_ **(L/g. min)**				**N**_**o**_ **(mg/L)**			**R** ^**2**^			**K**_**YN**_ **(min** ^**−1**^ **)**		**Ʈ (min)**
Sr²⁺																														
1 cm			**5 mL/min**			0.99		0.00117					15.21		0.90				0.00064		26705.6					0.99		0.0117		608.5
2 cm			**5 mL/min**			0.99		0.00105					9.38		0.95				0.00074		14236.1					0.99		0.0105		748.3
3 cm			**5 mL/min**			0.99		0.00099					7.23		0.97				0.00083		10039.7					0.99		0.0099		860.3
V⁵⁺																														
1 cm		**5 mL/min**				0.98				0.00126		14.56				0.89			0.00068		26272.3					0.98		0.0126		582.6
2 cm		**5 mL/min**				0.98				0.00110		8.74				0.95			0.00069		14072.4					0.98		0.0105		699.3
3 cm		**5 mL/min**				0.98				0.00097		6.56				0.97			0.00072		9803.4					0.98		0.0097		788.3
Rb⁺																														
1 cm		**5 mL/min**				0.97				0.00119		13.06				0.89			0.00052		26298.1					0.97		0.0119		522.4
2 cm		**5 mL/min**				0.98				0.00105		8.01				0.95			0.00063		13773.8					0.98		0.0105		640.8
3 cm		**5 mL/min**				0.97				0.00104		5.95				0.96			0.00071		9487.8					0.97		0.0104		714.3

#### Dynamic modelling of the column

This study employed three well-established kinetic models—Thomas, Adams-Bohart, and Yoon-Nelson—to analyze the dynamic adsorption behavior and performance of the MCM/talc-based column system. These models were utilized to predict breakthrough curves and assess the adsorption efficiency of the fixed-bed system. The Adams-Bohart and Yoon-Nelson models specifically focused on evaluating the column’s breakthrough characteristics and its affinity for dissolved pollutants during the initial phase of the decontamination process^92,94^. The parameters derived from these models offered valuable insights into the practical performance of the column, including estimates of saturation concentration, bed adsorption capacity, and the time required to achieve 50% breakthrough. The kinetic properties of the MCM/talc bed were determined using nonlinear regression analysis, incorporating the mathematical formulations of the Thomas, Adams-Bohart, and Yoon-Nelson models (Eq. 11, Eq. 12, and Eq. 13, respectively). These models play a crucial role in elucidating the mechanisms underlying adsorption processes and in optimizing the operational performance of fixed-bed column systems.

11 $$\:\text{ln}(\frac{{C}_{o}}{{C}_{t}}-1)=\frac{{K}_{Th}{q}_{o}M}{F}-{K}_{Th}{C}_{o}t\:\:\:\:\:\:\:\:\:$$

12 $$\:\text{ln}(\frac{{C}_{t}}{{C}_{o}})={K}_{AB}{C}_{o}t-{K}_{AB}{N}_{o}\frac{Z}{{U}_{o}}\:\:\:\:\:\:\:\:\:\:\:$$

13 $$\:\text{ln}\left(\frac{{C}_{t}}{{C}_{o}{-C}_{t}}\right)={K}_{YN}t-\tau\:{K}_{YN}\:\:\:\:\:\:\:\:\:\:\:\:\:\:\:\:$$

The Thomas model symbols are: K_Th_ (rate constant), q_o_ (adsorption capacity), C_o_, and C_t_ (initial and effluent concentrations), M (adsorbent mass), F (flow rate), and t (time). The symbols in the Adams–Bohart model are: K_AB_ (kinetic constant), U_o_ (linear velocity), Z (bed depth), and N_o_ (saturation concentration). In the Yoon–Nelson model, K_YN_ is the rate constant (min⁻¹), and τ is the time required for 50% breakthrough (min).

##### Thomas model

The Thomas model posits that the adsorption of the metal ions Sr²⁺, V⁵⁺, and Rb⁺ onto the MCM/talc fixed bed adheres to second-order kinetic principles. This model assumes reversible adsorption processes that align with Langmuir equilibrium behavior while neglecting the influences of axial dispersion and both internal and external diffusion limitations^95,96^. The determination coefficient (R²) values obtained under varying bed thicknesses demonstrate strong agreement between the experimental data and the theoretical predictions of the Thomas model (Fig. 9D–F; Table 2). Notably, as the bed thickness increases from 1 cm to 3 cm, the equilibrium adsorption capacities of the MCM/talc for the five metals exhibit a gradual decline.

##### Adams-Bohart model

The Adams-Bohart model provides a framework for analyzing the transport dynamics of metal ions through the MCM/talc fixed bed. This model assumes constant adsorption capacities for Sr²⁺, V⁵⁺, and Rb⁺, describing adsorption as a stepwise isotherm-based mechanism^97^. The experimental breakthrough data align closely with the Adams-Bohart model across various MCM/talc bed thicknesses and flow rates (Fig. 9G–I; Table 2). According to the model’s predictions, the saturation concentration (*N*_o_) of the metal ions decreases consistently with increasing bed thickness (Table 2). These observations underscore the applicability of the Adams-Bohart model in capturing the adsorption dynamics within the fixed-bed system.

##### Yoon-Nelson model

The Yoon-Nelson model is utilized to predict the time required for the MCM/talc fixed bed to reach 50% saturation and to describe the adsorption kinetics under specific operating conditions^93^. The adsorption kinetics governed by this model are directly influenced by the breakthrough behavior of the MCM/talc bed, determining the uptake rates of the metal ions^92^. High R² values confirm the strong agreement between the experimental data and the Yoon-Nelson model (Fig. 9J–L; Table 2). Model predictions indicate that for a 3 cm bed thickness, 50% saturation is achieved after 860.3 min for Sr²⁺, 788.3 min for V⁵⁺, and 714.3 min for Rb⁺. These findings suggest that increasing the bed thickness significantly enhances the operational lifetime of the column (Table 2). Adjustments to bed thickness and other operational parameters can lead to notable improvements in the system’s decontamination efficiency and processing capacity for large solution volumes.

### Realistic remediation of groundwater in Siwa Oasis

**Fig. 10:**
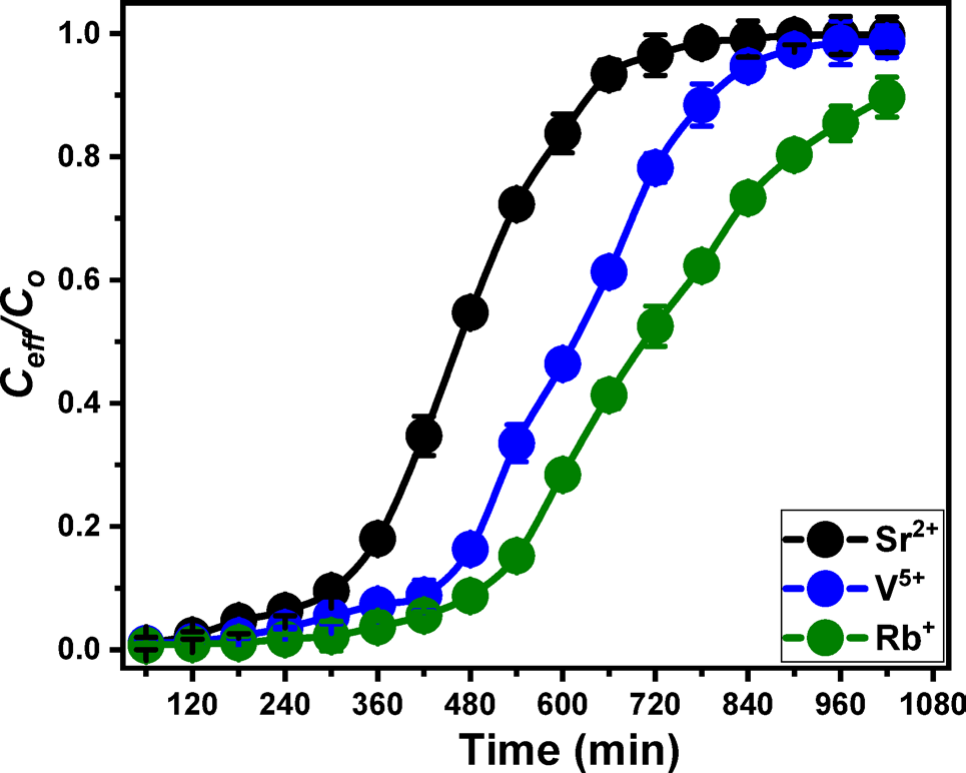
The breakthrough curves for the realistic adsorption of Sr²⁺, V⁵⁺, and Rb⁺ from groundwater in Siwa Oasis using MCM/talc bed.

A 3 cm MCM/talc bed was utilized for the practical removal of Sr²⁺, V⁵⁺, and Rb⁺ from groundwater samples collected from the Siwa Oasis. The experiments were conducted under controlled conditions, including a flow rate of 5 mL/min, pH of 6, and a total operational time of 840 min. The groundwater sample analyzed was representative of various wells in the region, with initial concentrations of Sr²⁺ (10.3 mg/L), V⁵⁺ (4.8 mg/L), and Rb⁺ (1.7 mg/L). The findings demonstrated the MCM/talc bed’s high efficacy in removing these metals, even after processing approximately 5 L of groundwater (Fig. 10; Table 2). Breakthrough times were recorded as 300 min for Sr²⁺, 420 min for V⁵⁺, and 480 min for Rb⁺, while exhaustion times were 720 min, 840 min, and 1020 min. Removal efficiencies of 42.7% for Sr²⁺, 56.3% for V⁵⁺, and 67.4% for Rb⁺ were achieved (Table 2). These variations in removal efficiencies compared to synthetic solutions can be attributed to the differing concentrations of metals in real groundwater and the presence of competing ions and anions, such as total hardness, phosphate, and nitrate, which influence the adsorption process. The total metal ions introduced into the column (M_total_​) were 472.7 mg for Sr²⁺, 220.3 mg for V⁵⁺, and 78.03 mg for Rb⁺, while the total adsorbed ions (Q_total_) were 101.7 mg/g, 78.4 mg/g, and 39.3 mg/g, respectively (Table 2). After treatment, the metal concentrations in the effluent were reduced to 5.9 mg/L for Sr²⁺, 2.1 mg/L for V⁵⁺, and 0.55 mg/L for Rb⁺. After introducing the treated water to second cycle of remediation using the fresh MCM/talc bed, the concentration declined significantly to 1.3 mg/L, 0.46 mg/L, and 0.03 mg/L for Sr²⁺, V⁵⁺, and Rb⁺ matching most of the environmental and health standards. These results highlight the effectiveness of the MCM/talc bed in removing toxic metal ions from groundwater, aligning with recommended safety limits. Enhanced performance can be achieved by increasing the bed thickness beyond 3 cm or employing a dual-bed system.

### Cost-effectiveness evaluation

The economic viability of the MCM/talc composite is a critical factor in determining its suitability for real-world water treatment applications. The cost-effectiveness of the developed adsorbent arises from its low-cost raw materials, simple synthesis process, and high performance across multiple cycles of use. The primary precursor, talc, is an abundant and inexpensive natural mineral, widely available in various regions, including Egypt. The synthesis process employs cost-efficient and commercially available reagents such as cetyltrimethylammonium bromide (CTAB) and ammonium hydroxide, without the need for high-temperature calcination or pressure-intensive equipment, thereby minimizing energy consumption and operational costs. In addition to low synthesis costs, the MCM/talc composite demonstrates high adsorption capacities for Sr²⁺, V⁵⁺, and Rb⁺ ions, which directly reduces the volume of adsorbent needed per treatment cycle. The column studies confirmed the material’s capacity for multiple treatment cycles with only modest decreases in efficiency, underlining its reusability. These features collectively contribute to lower lifecycle costs in comparison to conventional adsorbents. Furthermore, the ability to process real groundwater samples from the Siwa Oasis to concentrations within international safety limits using a small-volume fixed-bed system underscores its practical potential in rural and economically challenged regions. Overall, the combination of inexpensive raw materials, scalable synthesis, high adsorption performance, and reusability highlights the cost-effective nature of the MCM/talc composite as a viable option for large-scale environmental remediation.

## Conclusions

The integration of mesoporous silica (MCM-41) with exfoliated talc nanosheets resulted in a novel hybrid adsorbent with a well-defined pore structure (9.6 nm) and a surface area of 13.6 m²/g, facilitating improved ion accessibility and fast adsorption kinetics. Batch adsorption studies demonstrated high maximum adsorption capacities, with Langmuir Q_max_ values of 226.1 mg/g for Sr²⁺, 185.6 mg/g for V⁵⁺, and 136.1 mg/g for Rb⁺. These results were further supported by statistical physics modeling, which yielded saturation capacities (Q_sat_) in close agreement and confirmed a multi-ionic vertical stacking mechanism (*n* > 2). The low adsorption energy values (− 7.47 to − 4.53 kJ/mol) indicated a physisorption-dominated process, suggesting favorable regeneration potential. Continuous-flow fixed-bed column experiments validated the practical applicability of the material, achieving dynamic removal efficiencies of 79.7% (Sr²⁺), 73.4% (V⁵⁺), and 68.6% (Rb⁺). Breakthrough data fitted well with Thomas, Yoon–Nelson, and Adams–Bohart models, confirming the system’s predictive reliability. Application to real groundwater samples from the Siwa Oasis in Egypt demonstrated effective contaminant removal, with post-treatment concentrations reduced to 1.3 mg/L (Sr²⁺), 0.46 mg/L (V⁵⁺), and 0.03 mg/L (Rb⁺)—falling within or approaching international drinking water standards. Overall, the MCM-41/talc composite exhibits strong potential for both the remediation of hazardous metal ions and the recovery of valuable elements, particularly in water-scarce regions affected by natural or anthropogenic contamination.

### Recommendation

Future investigations will incorporate X-ray photoelectron spectroscopy (XPS) to directly assess surface elemental states and binding energy shifts. This will provide additional confirmation of the interaction pathways between metal ions and the functional groups of the Mg-MCM-41/talc composite.

## Electronic supplementary material

Below is the link to the electronic supplementary material.

Supplementary Material 1

## Data Availability

The data will be available up on request to corresponding author.

## Abbreviations

Q_e_: Equilibrium adsorption capacity (mg/g)
C_o_: Initial metal ion concentration (mg/L)
C_e_: Equilibrium metal ion concentration (mg/L)
V: Volume of solution (mL)
M: Mass of adsorbent (g)
Q_total_: Total amount of adsorbed ions (mg)
M_total_: Total introduced ions (mg)
Q_eq_: Column equilibrium capacity (mg/g)
C_eff_: Effluent concentration (mg/L)
C_ad_: Amount of adsorbate adsorbed (mg/g)
Q and F: Flow rate (mL/min)
t_total_: Total operation time (min)
A: Cross-sectional area
Z: Bed depth
X: Mass of MCM/talc adsorbent (g)
R^2^: Coefficient of determination
χ^2^: Chi-square value
RMSE: Root mean square error
Q_max_: Maximum adsorption capacity from Langmuir model (mg/g)
K_Th_: Thomas model rate constant (L/mg·h)
K_AB_: Adams–Bohart rate constant (L/g·min)
N_o_: Saturation concentration in Adams–Bohart model (mg/L)
K_YN_: Yoon–Nelson rate constant (min⁻¹)
τ: Time required for 50% breakthrough (min)
U₀: Initial velocity of the influent solution (m/h)
Q_sat_: Saturation adsorption capacity from statistical physics model (mg/g)
n: Number of ions per active site
N_m_: Density of receptor sites (mg/g)
C_1/2_: Concentration at half saturation (mg/L)
ΔE: Adsorption energy (kJ/mol)
β: D–R model constant related to mean free energy (mol²/kJ²)
Q_m_: Maximum adsorption capacity from D–R model (mg/g)
K_F_: Freundlich constant related to adsorption capacity
1/n: Freundlich intensity constant
R_L_: Langmuir separation factor
k_1_: Pseudo-first-order rate constant (min⁻¹)
k_2_: Pseudo-second-order rate constant (g/mg·min)
k_id_: Intraparticle diffusion rate constant (mg/g·min⁰·⁵)
T: Absolute temperature (K)
R: Universal gas constant (8.314 J/mol·K)
Q_t_: Adsorption capacity at time t (mg/g)
t: Contact time (min)
k_d_: Desorption rate constant
