# Supplementary material for: Efficient removal of Sr²⁺, V⁵⁺, and Rb⁺ ions from groundwater using a hybrid Mg-MCM-41/Talc composite; Siwa Oasis in Egypt as case study
Source: Sci Rep. 2025 Jul 12;15:25191. doi: 10.1038/s41598-025-09553-3 (PMC12255759; doi:10.1038/s41598-025-09553-3)
Supplement: Supplementary file 1 — Supplementary Material 1 [file 41598_2025_9553_MOESM1_ESM.docx]

**Table S1.** Nonlinear equations of kinetic, classic isotherm, and advanced isotherm models

| Kinetic models | | |
| --- | --- | --- |
| Model | **Equation** | **Parameters** |
| Pseudo-first-order | $Q_{t}=Q_{e} (1-e^{{-k}_{1}.t})$ | Q_t_ (mg/g) is the adsorbed ions at time (t), and K_1_ is the rate constant of the first-order adsorption (1/min) |
| Pseudo-second-order | $Q_{t}=\frac{Q_{e}^{2}k_{2}t}{1+Q_{e}k_{2}t}$ | Qe is the quantity of adsorbed ions after equilibration (mg/g), and K_2_ is the model rate constant (g/mg min). |
| Classic Isotherm models | | |
| Model | **Equation** | **Parameters** |
| Langmuir | $Q_{e}=\frac{Q_{max} bC_{e}}{(1+bC_{e})}$ | *C_e_* is the rest ions concentrations (mg/L), *Q_max_* is the theoritical maximum adsorption capacity (mg/g), and *b* is the Langmuir constant (L/mg) |
| Freundlich | $Q_{e}=K_{f}C_{e}^{1/n}$ | K_F_ (mg/g) is the constant of Freundlich model related to the adsorption capacity and n is the constant of Freundlich model related to the adsorption intensities |
| Dubinin–Radushkevich | $Q_{e}=Q_{m}e^{-\betaɛ^{2}}$ | β (mol^2^/KJ^2^) is the D-R constant, ɛ (KJ^2^/mol^2^) is the polanyil potential, and Q_m_ is the adsorption capacity (mg/g) |
| Advanced isotherm models | | |
| Model | **Equation** | **Parameters** |
| Monolayer model with one energy site (Model 1) | $Q=nN_{o} =\frac{nN_{M}}{1+{(\frac{C1/2}{C})}^{n}}=\frac{Q_{o}}{1+{(\frac{C1/2}{C})}^{n}}$ | Q is the adsorbed quantities in mg/g  n is the number of adsorbed ion per site  Nm is the density of the effective receptor sites (mg/g)  Q_o_ is the adsorption capacity at the saturation state in mg/g  C1/2 is the concentration of the ions at half saturation stage in mg/L  C1 and C2 are the concentrations of the ions at the half saturation stage for the first active sites and the second active sites, respectively  n1 and n2 are the adsorbed ions per site for the first active sites and the second active sites, respectively |
| Monolayer model with two energy sites (Model 2) | $Q=\frac{n_{1}N_{1M}}{1+{(\frac{C_{1}}{C})}^{n_{1}}}+\frac{n_{2}N_{2M}}{1+{(\frac{C_{2}}{C})}^{n_{2}}}$ |  |
| Double layer model with one energy site (Model 3) | $Q=Q_{o}\frac{({\frac{C}{C1/2})}^{n}+2({\frac{C}{C1/2})}^{2n}}{1+({\frac{C}{C1/2})}^{n}+({\frac{C}{C1/2})}^{2n}}$ |  |
| Double layer model with two energy sites (Model 3) | $Q=Q_{o}\frac{({\frac{C}{C1})}^{n}+2({\frac{C}{C2})}^{2n}}{1+({\frac{C}{C1})}^{n}+({\frac{C}{C2})}^{2n}}$ |  |


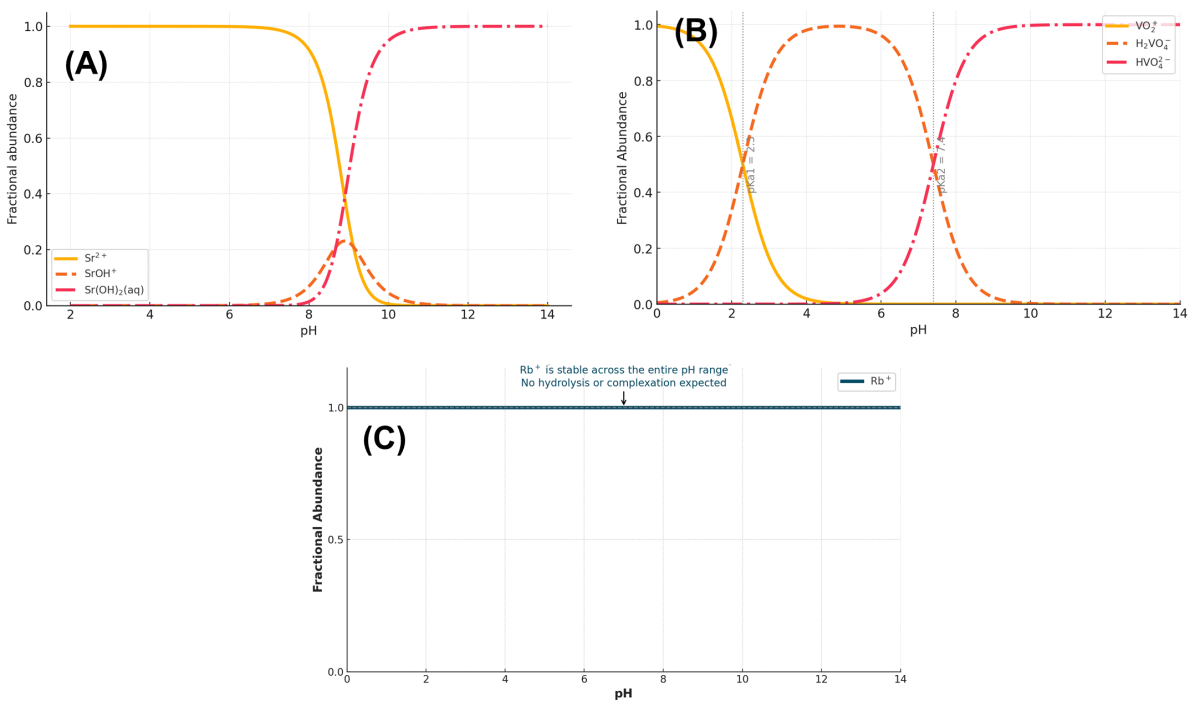


**Fig S1.** The speciation diagram of the studied metal ions ate different pH (A) Sr²⁺, (B) V⁵⁺, and (C) Rb⁺


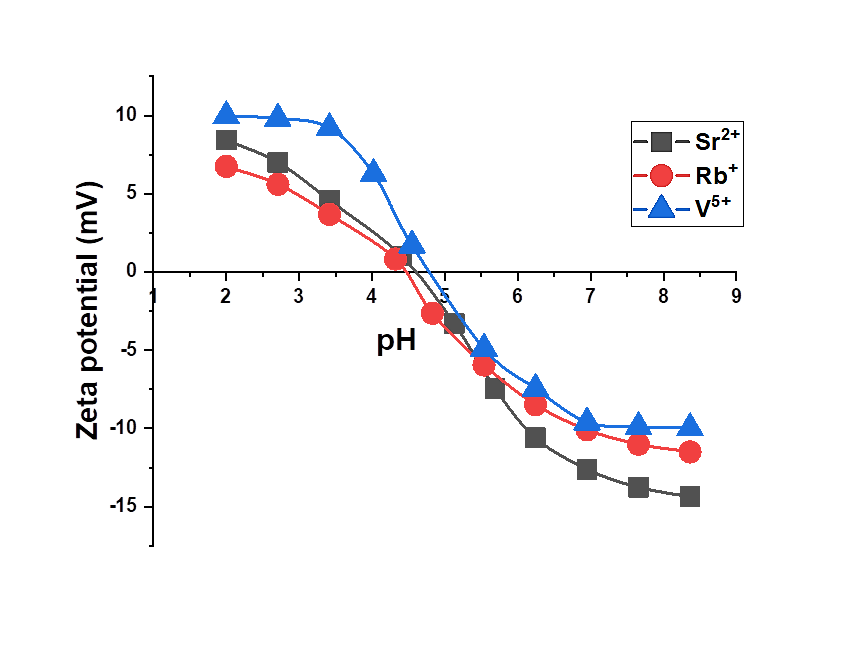


**Fig. S2.** Symbolic zeta potential curves of the MCM/talc and the specific pHₚzc values
